# Lysine-specific histone demethylase 1a regulates nephron development and long-term transcriptional programming

**DOI:** 10.1172/jci.insight.190283

**Published:** 2026-03-09

**Authors:** Nicola Wanner, Julia Keller, Nastassia Liaukouskaya, Geoffroy Andrieux, Sandra D. Laufer, Manuel Rogg, Tillmann Bork, Wei Liang, Fabian Braun, Fabian Haas, Milagros N. Wong, Victor G. Puelles, Sydney E. Gies, Charlotte Meyer, Melanie Boerries, Martin Helmstädter, Oliver Kretz, Iris Hild, Eric Metzger, Roland Schüle, Wibke Bechtel-Walz, Tobias B. Huber

**Affiliations:** 1 III. Department of Medicine and; 2 Hamburg Center for Kidney Health (HCKH), University Medical Center Hamburg-Eppendorf, Hamburg, Germany.; 3 Department of Medicine IV, Faculty of Medicine, University of Freiburg, Germany.; 4 Faculty of Biology, Albert Ludwigs University of Freiburg, Freiburg, Germany.; 5 Institute of Medical Bioinformatics and Systems Medicine and; 6 Institute of Surgical Pathology, Faculty of Medicine, Medical Center - University of Freiburg, Freiburg, Germany.; 7 Division of Nephrology, Renmin Hospital of Wuhan University, Wuhan, China.; 8 Martin Zeitz Center for Rare Diseases, University Medical Center Hamburg-Eppendorf (UKE), Hamburg, Germany.; 9 Department of Clinical Medicine, Aarhus University, Aarhus, Denmark.; 10 Department of Pathology, Aarhus University Hospital, Aarhus, Denmark.; 11 German Cancer Consortium (DKTK), Partner site Freiburg, a partnership between DKFZ and Medical Center, University of Freiburg, Freiburg, Germany.; 12 EMcore, Renal Division, Department of Medicine, University Hospital Freiburg, University Faculty of Medicine, Freiburg, Germany.; 13 UKE Electron Microscopy Core Facility, University Medical Center Hamburg-Eppendorf (UKE), Hamburg, Germany.; 14 Department of Urology, University Freiburg Medical Center, Freiburg, Germany.

**Keywords:** Development, Nephrology, Chronic kidney disease, Epigenetics

## Abstract

Low nephron endowment constitutes a risk factor for hypertension and renal disease. Epigenetic regulation is crucial for nephron progenitor cell differentiation, affecting nephron number and renal function. The role of many epigenetic modulators, such as Lysine-specific histone demethylase 1a (LSD1 or KDM1A), remains unclear. We used *Kdm1a*-KO mice to demonstrate that *Kdm1a* depletion in nephron progenitor cells results in reduced kidney size in neonates and led to glomerulosclerosis, proteinuria, and renal cysts in adults. Notably, *Kdm1a* deletion in podocytes or tubular cells did not replicate these effects. CRISPR/Cas9-mediated *KDM1A* deletion in human kidney organoids caused cyst formation and altered gene expression, with snRNA-seq revealing downregulation of podocyte genes and upregulation of metabolic genes. The presence of noncoding RNAs indicated roles in cell proliferation. Our study reveals the critical role of *Kdm1a* function in nephron development and highlights its affect on transcriptional programming for long-term renal function and susceptibility to cyst formation.

## Abstract 2

LSD1 regulates kidney development, and its dysfunction disrupts key kidney cells, leading to cyst formation. Targeting LSD1 could offer new treatments for kidney diseases.

## Graphical abstract

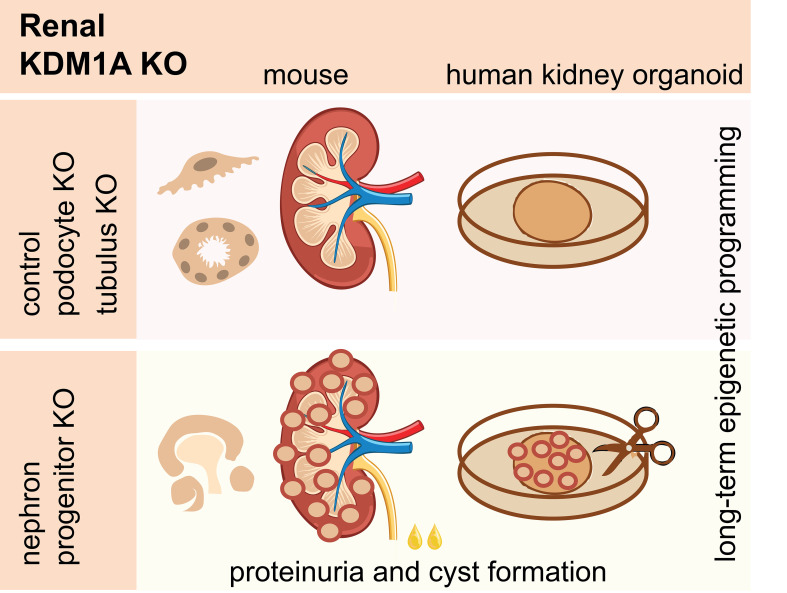

## Introduction

Despite advances in the medical field, kidney disease affects millions of people world-wide with increasing numbers, thus posing a global health challenge. Progression of kidney disease often leads to end-stage renal disease, necessitating dialysis or kidney transplantation. Apart from manifesting as a symptom alongside other illnesses, such as cardiovascular disease, renal dysfunction can arise due to genetic predispositions or environmental factors. For instance, proper renal development substantially influences kidney functionality and longevity. The number of nephrons per kidney is determined during nephrogenesis, with a low nephron endowment increasing the risk of hypertension and progression of renal disease (1, 2). Low birth weight (3, 4) as well as metabolic influences like low protein diet (5), maternal diabetes (6), or obesity (7, 8) correlate with reduced nephron number and long-term functional deficits.

Within the last decades, the principles of renal development driving cellular differentiation, renal morphogenesis, and patterning have been unraveled (9). Reciprocal interactions between ureteric bud tips and the cap mesenchyme (CM) induce ureter branching and formation of functional nephrons (9). The CM, a pool of multipotent and self-renewing progenitor cells, forms around the ureteric bud tips and induces formation of nephrons by condensation of the mesenchymal cells into epithelialized and polarized renal vesicles, which then morph into comma-shaped and s-shaped bodies, giving rise to glomeruli and tubules (10–13). While the intricate molecular signaling networks during these processes are well characterized, the effect of epigenetic regulation on nephrogenesis is still mostly unknown (14). Recently, our group demonstrated that DNA methyltransferase 1 (DNMT1) controls nephron progenitor cell renewal as well as differentiation (15). A comparative analysis of the epigenetic state revealed differences in the global level of histone methylation between self-renewing cells (Six2^hi^) and induced cells (Six2^lo^) of the CM, indicating major epigenetic processes occurring during nephron induction (16, 17). Further studies showed differential methylation in promotor regions of stemness marker, while silenced cell-specific genes are decreased in H3K4 trimethylation. In contrast, induced CM shows activity of epithelial marker in conjunction with high H3K4me3 and loss of H3K9me2 signature. Moreover, loss of histone methylation in stemness genes was found (17).

The lysine-specific histone demethylase 1a (*Lsd1*, gene name *Kdm1a*) has been shown to be crucial for differentiation of embryonic stem cells (ESCs) by simultaneously demethylating and suppressing ESC-specific genes (18) and activating cell-specific genes (19). KDM1A is composed of an N-terminal SWIRM (small alpha helical) domain, a C-terminal AOL (amine oxidase like) domain with a flavin adenine dinucleotide–binding (FAD-binding) site and the catalytic center, as well as a central tower domain (20, 21). KDM1A specifically demethylates mono- and dimethylated H3K4 and H3K9 (22) but also acts as scaffolding protein to several interaction partners (23–26). While the contribution of KDM1A has been described in several developmental processes, its role for kidney development and function is still unknown. In this study, we examine the effect of KDM1A in prenatal renal programming, nephron formation, and renal function.

## Results

### KDM1A is expressed in early nephron development.

**Figure 1:**
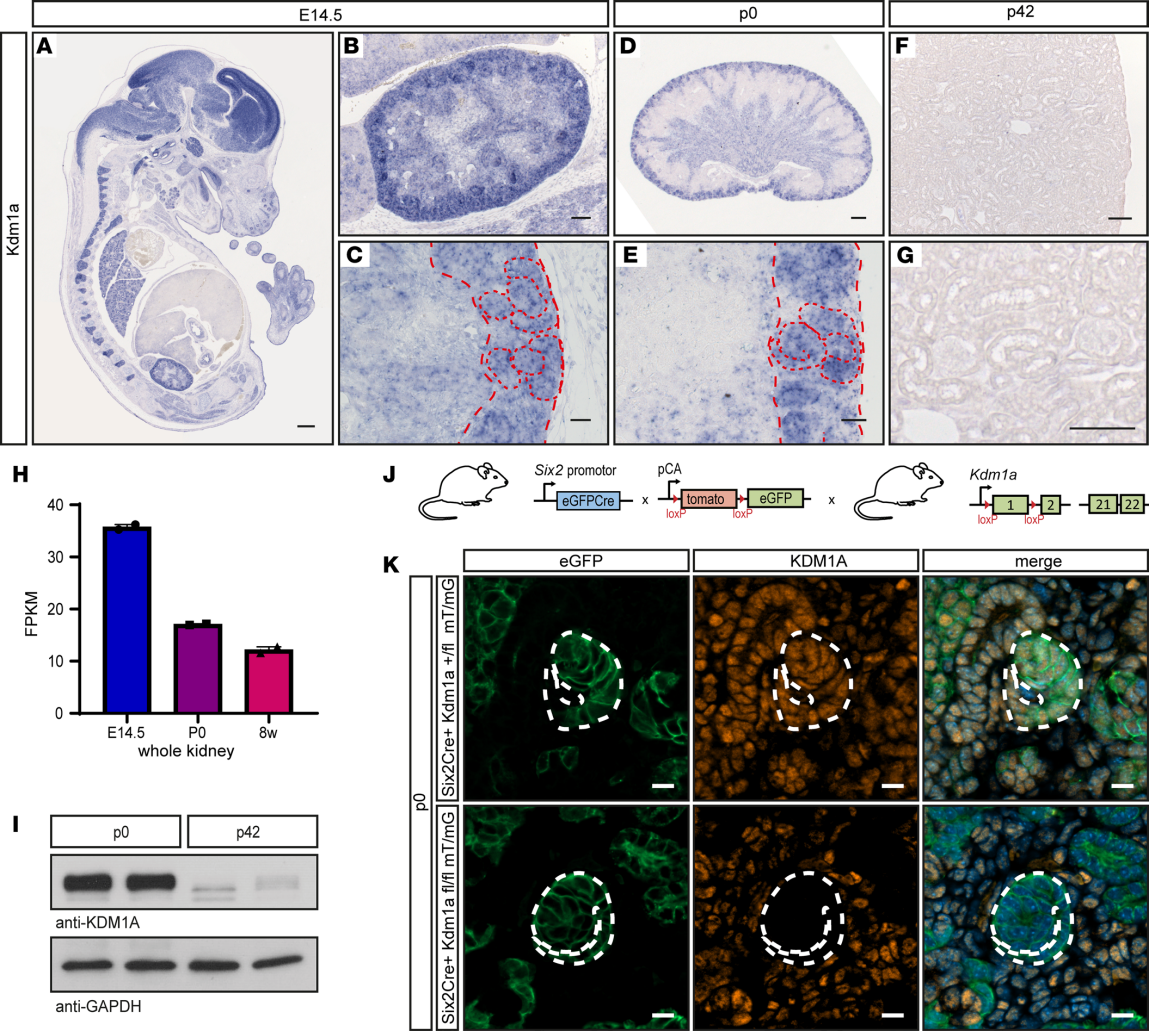
*Kdm1a* is expressed in the nephrogenic zone of the developing kidney. (**A**) Expression pattern of *Kdm1a* in WT embryo at E14.5 (500 μm) and (**B**) WT kidney at E14.5 (100 μm) and (**C**) magnified view (30 μm), (**D**) p0 (200 μm) and (**E**) magnified view (30 μm) shows accumulation of mRNA in the nephrogenic zone and early nephrons during renal development of WT mice (red dotted lines) using ISH. (**F**) In the adult kidney, no mRNA expression is detectable (scale bar: 100 μm), (**G**) magnified view (scale bar: 100 μm). (**H**) Gene expression in whole kidney ENCODE data sets shows decrease of *Kdm1a* expression during aging. FPKM, Fragments Per Kilobase of transcript per Million mapped reads. (**I**) Western blot in whole kidney tissue shows loss of KDM1A protein in the adult kidney. (**J**) Schematic of conditional *Kdm1a*-KO reporter mouse model. (**K**) Proof of conditional KO by immunofluorescence staining of GFP transgene (green) and KDM1A (orange) on p0 kidneys of heterozygous and KO. Scale bar: 10 μm.

KDM1A has previously been shown to be crucial for many developmental processes (18, 27). However, the role of KDM1A for kidney development is still unknown. We therefore analyzed *Kdm1a* expression in embryonic mouse tissue. In situ hybridization shows *Kdm1a* expression at E14.5 in the lung and brain as well as in the kidney (Figure 1A and Supplemental Figures 1 and 2; supplemental material available online with this article; https://doi.org/10.1172/jci.insight.190283DS1). *Kdm1a* expression was predominant in the nephrogenic zone and early nephron structures at E14.5 and postnatal day 0 (p0) developmental stages (Figure 1, B and C’). In contrast, almost no *Kdm1a* expression was detected in adult renal tissue (Figure 1D). Whole-kidney RNA and Western blot analyses confirmed *Kdm1a* expression in embryonic and p0 kidneys and downregulation in the adult renal tissue (Figure 1, E and F).

To study *Kdm1a* function in the kidney, we bred KO mice with a conditional deletion of the first exon of the *Kdm1a* gene (*Kdm1a^fl/fl^*) (28) using the Six2-TGC^tg^ Tg(Six2-EGFP/cre)1Amc/J (*Six2*Cre) line (29) targeting renal cap mesenchymal cells as well as their descendants (Figure 1G and Supplemental Figure 3). For permanent tracing of the cells, the reporter mouse strain Gt(ROSA)26Sortm4(ACTB-tdTomato,-EGFP)Luo/J (*tomato^fl/fl^*) (30) was used. The KO of KDM1A was confirmed by immunofluorescence analysis of KDM1A and EGFP on p0 kidneys. While control kidneys display *Kdm1a* expression in almost all cells of the nephrogenic zone including EGFP^+^ cells (Figure 1H, upper panel), KO kidneys showed no KDM1A protein signal in the developing renal structures, such as comma-shaped bodies (Figure 1H and Supplemental Figure 4). However, remaining KDM1A signal can still be seen in the surrounding stromal cells of the kidney (Figure 1H). RNA-seq of E14.5 CM showed minimal transcriptional changes and unchanged NPC marker expression, consistent with unchanged histone modification patterns by IF (Supplemental Figures 5–7)

### Conditional depletion of KDM1A in nephron progenitor cells leads to mild kidney hypoplasia and proteinuria at birth.

**Figure 2:**
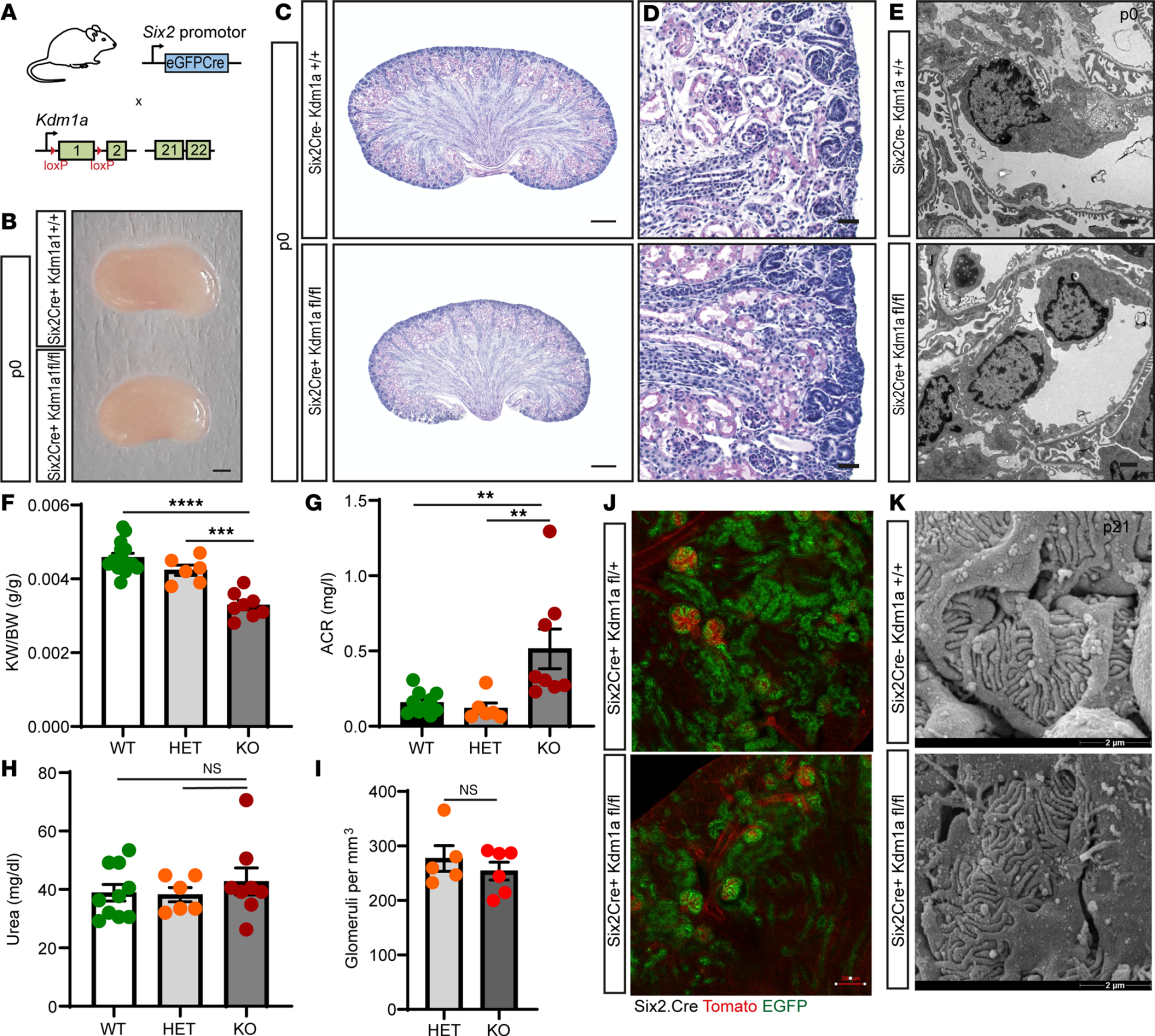
Conditional depletion of KDM1A leads to mild proteinuria and kidney hypoplasia at birth. (**A**) Schematic of conditional *Kdm1a*-KO mouse model. (**B**) Macroscopic view of WT and KO kidneys (scale bar: 5 mm). (**C** and **D**) Periodic Acid Schiff staining of histological sections of WT and KO kidneys shows no structural changes upon conditional *Kdm1a* ablation (scale bars: 300 μm in **C** and 30 μm in **D**). (**E**) Transmission electron microscopy of WT and KO kidney displays slight abnormalities in foot process formation. Scale bar: 1 μm. (**F**) Kidney weight-to-body weight ratio shows reduction of kidney size (wt, *n* = 14; het, *n* = 6; ko, *n* = 8). (**G**) Albumin/creatinine ratio shows an increase in proteinuria in KO animals compared with WT and HET animals. (**H**) Blood urea levels are unaltered. One-way ANOVA with Dunnett’s correction for multiple comparisons. (**I**) Glomeruli per volume do not differ compared with HET and KO kidneys at p0. Unpaired 2-tailed Student’s *t* test. (**J**) Representative images from *z* stacks of optically cleared kidneys at p0. Scale bar: 100 μm. (**K**) Scanning electron microscopy images from 21-day-old kidneys show slightly irregular foot process effacements in KO kidneys. Scale bar: 2 μm. *P < 0.01. **P < 0.05.

*Kdm1a-*KO animals (Figure 2A) and their control littermates were born at a Mendelian ratio (Supplemental Figure 8A). Macroscopically, the *Kdm1a*-KO kidneys appeared smaller (Figure 2B). Histological analysis revealed no structural changes in KO nephrons (Figure 2, C and D), and transmission electron microscopy (TEM) showed mostly unaffected podocyte foot process arrangements (Figure 2E). However, some slight irregularities such as thin or even fused foot processes were detected in the absence of *Kdm1a* during nephrogenesis (Figure 2E). While the body weight of newborn KO mice did not differ from their control littermates, *Kdm1a-*KO kidneys were significantly reduced in weight compared with WT and heterozygous controls, which led to a significantly reduced kidney/body weight ratio (Figure 2F and Supplemental Figure 8, B and C). Urinary analysis revealed mild but significant increase of albumin/creatinine ratio (Figure 2G), while no increase of urea could be detected in *Kdm1a-*KO blood serum at this stage (Figure 2H). Using optical clearance and 3D imaging of kidney tissue from newborn KO and control mice, no change in nephron density was detected (Figure 2, I and J). At p21, only slight irregularities can be seen in podocyte foot processes (Figure 2K). Thus, while nephrogenesis per se is not affected, conditional loss of *Kdm1a* during nephrogenesis impairs renal growth and has a mild effect on the glomerular filtration barrier.

### Loss of KDM1A in nephron progenitor cells leads to proteinuria and cyst formation in the adult kidney.

**Figure 3:**
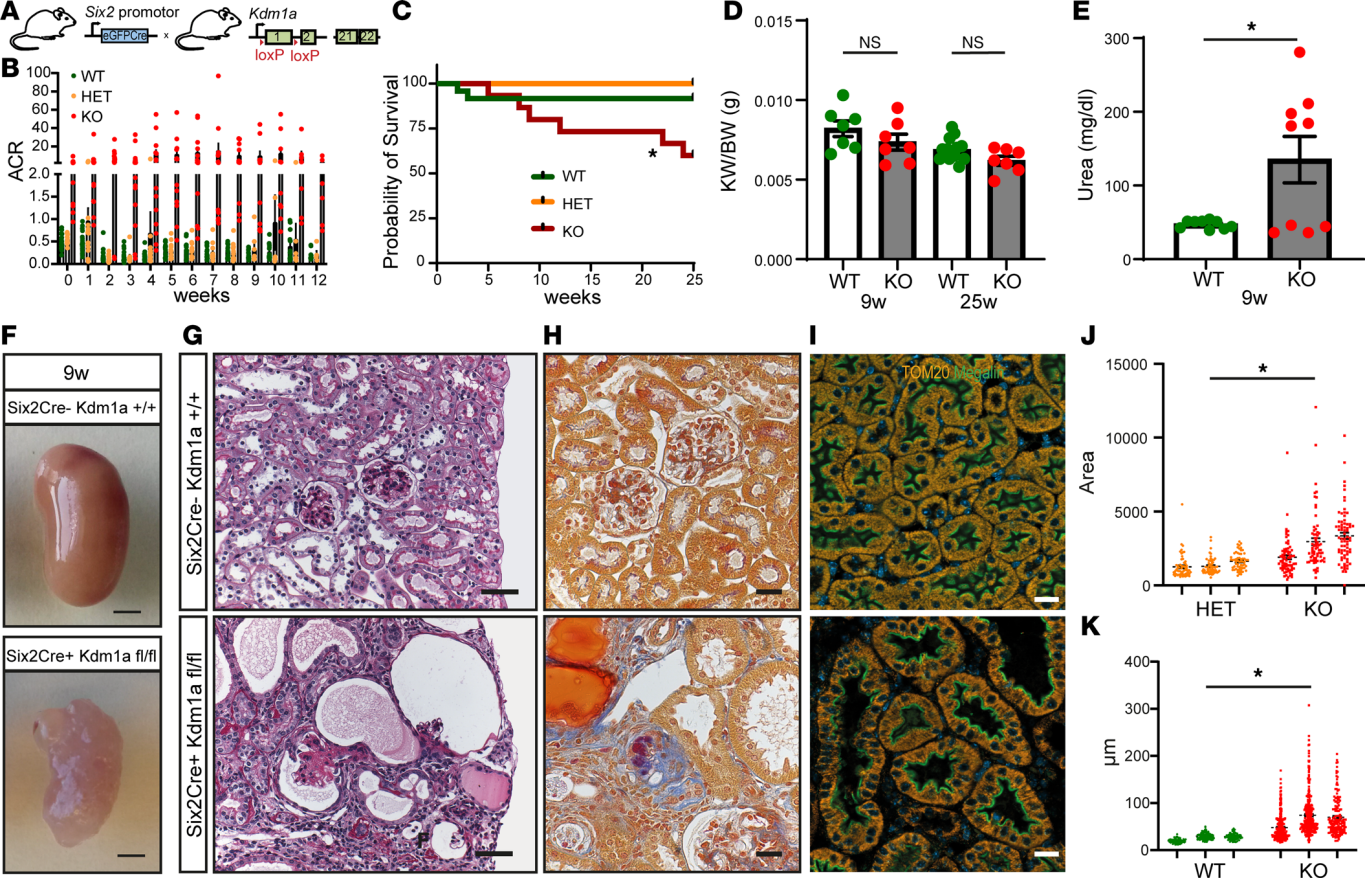
Loss of KDM1A during nephron formation induces massive changes in the adult kidney. (**A**) Schematic of the Six2Cre *Kdm1a* KO. (**B**) *Kdm1a* KO animals show high levels of proteinuria. (**C**) Kaplan-Meier curve of KDM1A KO animals vs control animals shows decreased survival. *P < 0.05. Log-rank test. (**D**) Kidney weight-to-body-weight ratio does not differ at 9 weeks between WT and KO animals (*n* = 7). (**E**) Urea levels are increased in some, but not all, of the KO animals (*n* = 9). (**F**) At macroscopic view, KO kidneys showed irregular surface and cysts compared with WT kidneys. Scale bar: 2 mm. (**G** and **H**) Histological analysis of WT and KO kidney displayed sclerotic glomeruli (scale bar: 50 μm) (**G**) as well as accumulation of collagen (scale bar: 25 μm) (**H**). (**I**) Tom20 (mitochondria) and Megalin (proximal tubules) stainings show dilations of proximal tubules in the KO animals. Scale bars: 20 μm. (**J** and **K**) Area of proximal tubules (arbitrary units, *n* = 3) (**J**) and diameter in μm of distal tubules (*n* = 3) are increased (**K**). *P < 0.05. Unpaired 2-tailed Student’s *t* test (**D**, **E**, **J**, and **K**).

*Kdm1a*-KO mice and control litter mates (Figure 3A) were further monitored. Analysis of proteinuria showed development of variably high levels of albumin/creatinine ratio over 12 weeks with all KO animals developing at least mild and some severe proteinuria (Figure 3B). Analysis over 25 weeks shows decreased survival in the *Kdm1a*-KO animals (Figure 3C). At 9 weeks and 25 weeks of age, no differences in kidney weight/body weight ratio can be detected (Figure 3D). Urea levels at 9 weeks show a split between *Kdm1a*-KO animals with normal urea levels and *Kdm1a*-KO animals with increased urea levels (Figure 3E). Kidneys from *Kdm1a*-KO mice at 9 weeks showed gross macroscopic changes with irregular renal surface and cystic appearances (Figure 3F and Supplemental Figure 9). Histological analysis revealed severe structural changes including sclerotic glomeruli, protein casts, and collagen-stained fibrotic tissue as well as dilated tubules (Figure 3, G–I, and Supplemental Figure 10). Quantification of dilated tubules show high variability in tubule size affecting proximal tubules (Figure 3J) and distal tubules (Figure 3K). Detailed analysis by TEM revealed hypertrophic podocytes with irregular primary and fused secondary foot processes of *Kdm1a*-KO kidneys, as well as accumulation of cell debris in urinary space (Supplemental Figure 8D). These data show a major effect of *Kdm1a* ablation in early nephrogenesis for structure and function of adult kidneys.

### Demethylase activity of KDM1A is required in nephrogenesis.

**Figure 4:**
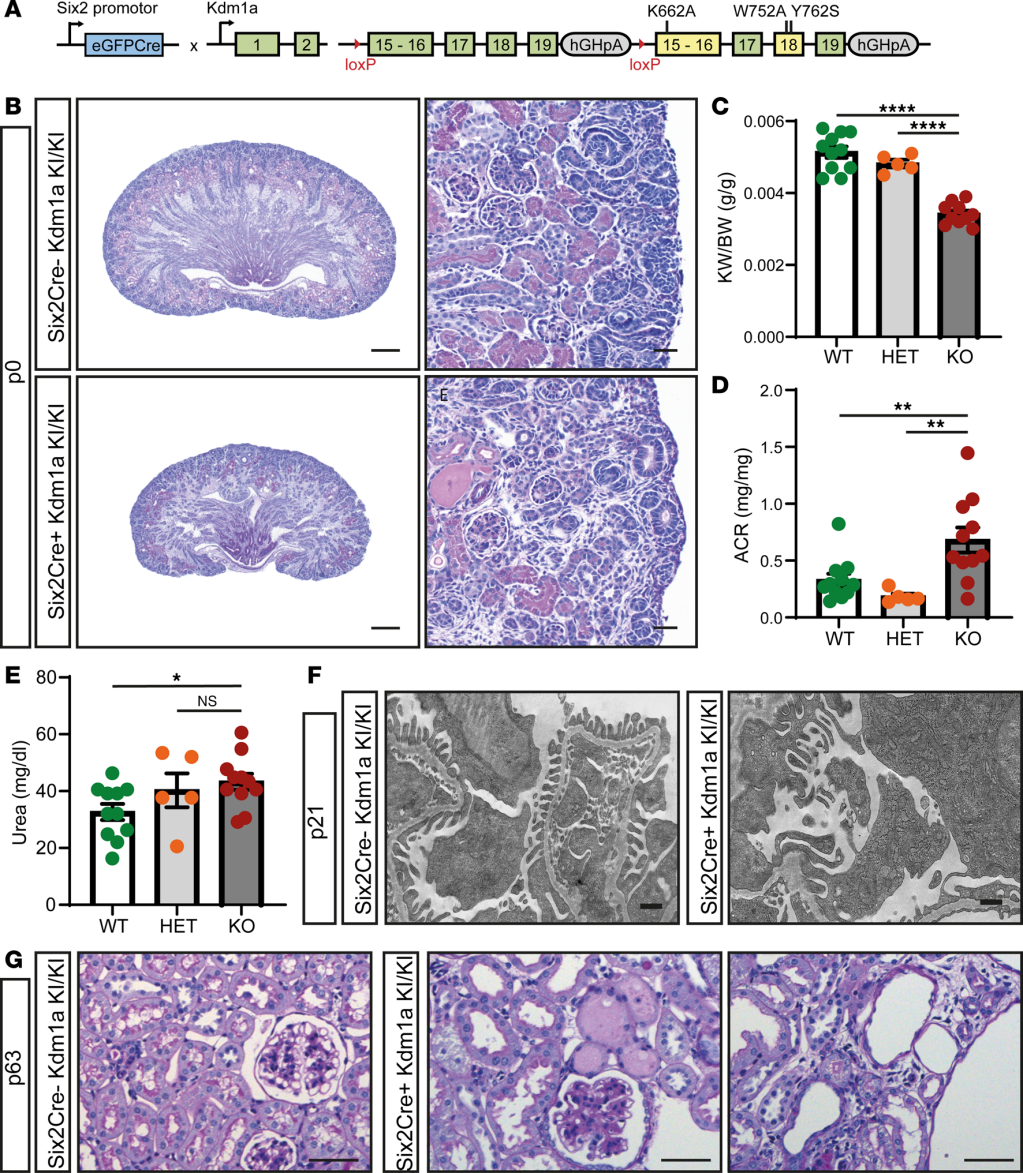
Demethylase activity of KDM1A is required for renal development. (**A**) Schematic of Six2Cre Kdm1a*^KI/KI^* mouse model. (**B**) Histological staining (PAS) of WT and KI kidney at p0 displayed no difference in tissue structure (scale bars: 300 μm [left] and 30 μm [right]). (**C**) Kidney/body weight ratiodemonstrated reduced cKI kidney weight at p0. ****P < 0.0001. (**D**) Albumin/creatinine ratio [mg/mg] in the urine indicates a slight dysfunction of KI kidneys. WT, *n* = 11; HET, *n* = 5; KO, *n* = 11. **P < 0.01. (**E**) Urea levels [mg/dL] in the blood serum in KI animals. *P < 0.05. One-way ANOVA with Dunnett’s correction for multiple comparisons. (**F**) Transmission electron microscopy reveals irregular foot processes at p21 in KI kidneys. Scale bar: 500 nm. (**G**) Histological sections at p63 demonstrate protein casts, glomerular sclerosis and dilated tubules in KI animals. Scale bar: 100 μm.

KDM1A not only functions as a histone demethylase but also serves as an interaction partner to many proteins (23–26). Therefore, we used a *Kdm1a–*knock-in (KI) (C57BL/6-Kdm1a^tm2931(K662A, W752A, Y762S)Arte^; hereafter referred to as *Kdm1a* KI) (31) mouse strain that substitutes the endogenous *Kdm1a* gene by an enzymatically inactive variant upon Cre expression (Figure 4A). Similar to the conditional KO mice, *Kdm1a* KI mice did not differ in body weight (*P* = 0.58) and showed no histological differences at p0 (Figure 4B), but a significant reduction in kidney/body weight ratio (Figure 4C) and increase of proteinuria (Figure 4D), while urea levels were only slightly altered (Figure 4E). At 3 weeks of age (p21), electron microscopy showed thin, irregular or even fused foot processes in the *Kdm1a* KI mice (Figure 4F). At 9 weeks (p63), protein casts, glomerular sclerosis and tubular dilations were visible in histological sections (Figure 4G). These findings correspond to the observed phenotype of the *Kdm1a*-KO kidneys and implicate a role of the histone demethylase function of KDM1A for renal development.

### KDM1A is dispensable in podocytes or proximal tubular cells.

**Figure 5:**
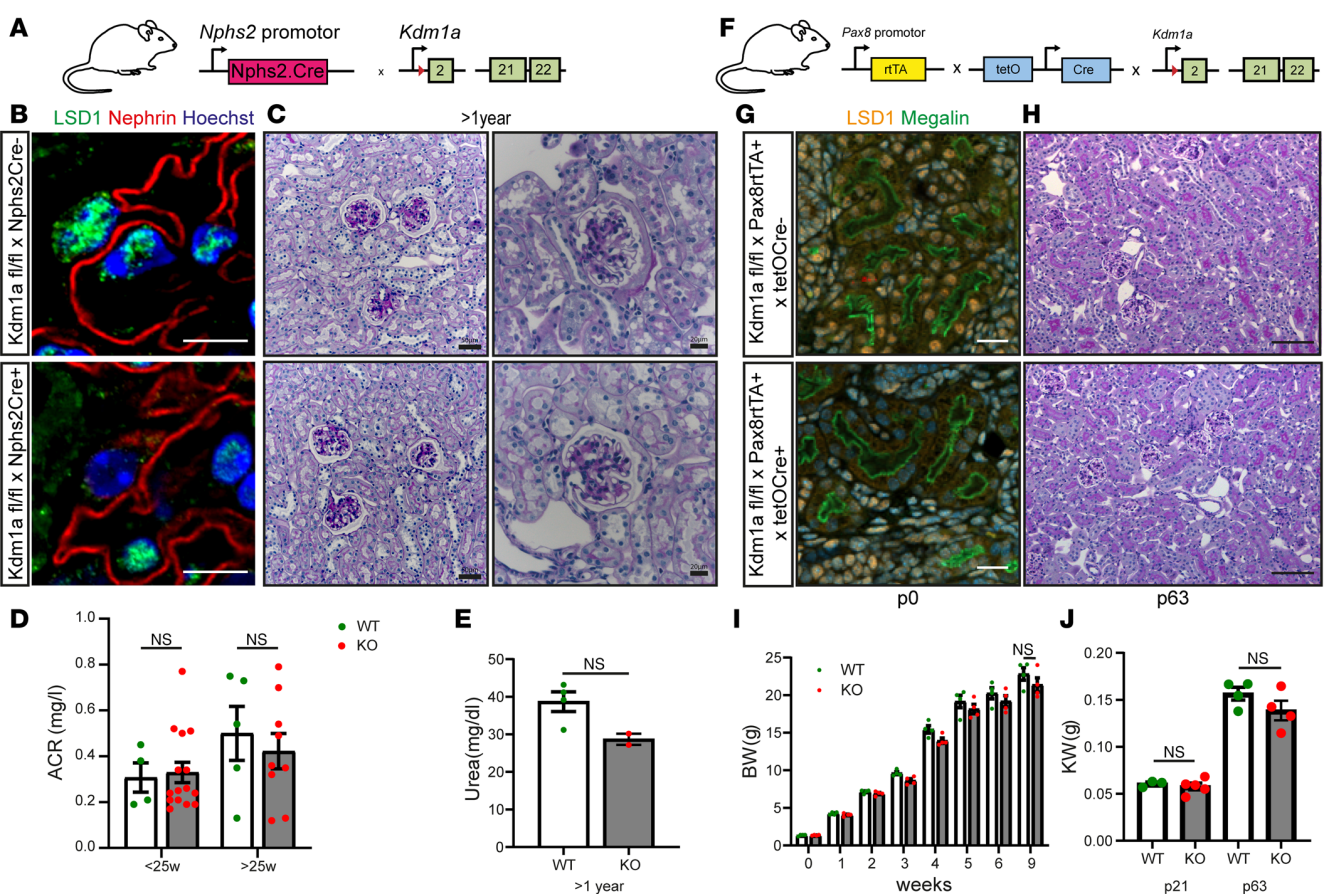
Specific loss of KDM1A in podocytes or proximal tubular cells does not impair kidney function. (**A**) Schematic of the conditional *NPHS2Cre Lsd1^fl/fl^* mouse model. (**B**) Antibody staining shows KDM1A in the Nephrin-positive podocytes in the WT, but not in Pod.Cre KO animals at 5 weeks. Scale bar: 10 μm. (**C**) Histological analysis (PAS) of WT and KO kidneys show no structural changes upon conditional *Kdm1a* ablation in podocytes. Scale bars: 50 μm (left) and 20 μm (right). (**D** and **E**) Albumin/creatinine ratio in the urine and urea levels in the blood serum at < 25 weeks, < 25 weeks, and < 1 year show no differences in the WT and podocyte KO animals. (**F**) Schematic of the Pax8rtTA tetOCre Kdm1a^fl/fl^ mouse model. (**G**) Antibody staining shows KDM1A in the Megalin-positive proximal tubules in the WT, but not in *Pax8.rtTA tetOCre* KO animals. Scale bar: 20 μm. (**H**) Histological analysis (PAS) of WT and KO kidney at p63 show no structural changes upon inducible *Kdm1a* ablation in proximal tubules. Scale bar: 200 μm. (**I** and **J**) Body weight and kidney weight of *Pax8.rtTA tetOCre* KO animals at p21 and p63 show no difference to WT animals. Unpaired 2-tailed Student’s *t* test was used.

To further examine the role of KDM1A for individual cellular compartments, we generated conditional KO mice targeting podocytes with *NPHS2.*Cre (Figure 5A). KO of *Kdm1a* was confirmed in immunofluorescence staining (Figure 5B). Histologically, no differences in tissue were visible in KO and control animals that were > 1 year old (Figure 5C). Albumin/creatinine levels did not differ in animals at various time points until > 1 year old (Figure 5D). Furthermore, no increase in urea levels were detectable (Figure 5E). To further analyze the role of KDM1A for the tubular compartment, inducible *Kdm1a*-KO mice were generated using the Pax8-coupled reverse tetracycline-dependent transactivator (*Pax8.*rtTA; tetO.Cre) line (Figure 5F). The KO was induced at E8.5 until birth and the KO confirmed by immunofluorescence (Figure 5G). At 9 weeks, the kidney tissue of the KO animals looks unaffected, and no cysts can be detected (Figure 5H). Neither body weight nor kidney weight is affected (Figure 5, I and J). These results indicate that KDM1A activity is crucial within a small time frame of early nephron development, but it is dispensable for podocyte and tubule differentiation at a later stage.

### Loss of KDM1A leads to altered mitochondrial function.

Several studies have shown KDM1A to be a key player in metabolic processes of different cell types (32–34), and TEM images at birth (p0) showed irregular mitochondria, mitophagy, and mitochondrial fusion in *Kdm1a*-deficient animals (Supplemental Figure 11). Therefore, the role of KDM1A for mitochondrial function of renal cells was analyzed. Primary nephron cells of WT and KO/KI mice at p0 were isolated and the mitochondrial respiration analyzed. In the *Kdm1a*-deficient cells, the basal as well as the maximum mitochondrial respiration of *Kdm1a*-deficient cells is higher compared with WT cells, while rates of ATP-linked respiration and proton leak do not differ significantly (Supplemental Figure 12A). *Kdm1a*-deficient cells show a slightly increased mitochondrial membrane potential (Supplemental Figure 12, B and C) but no increase in production of reactive oxygen species (Supplemental Figure 12D). Staining and quantification of mitochondria with both TOM20 and MitoTracker in vitro show increased intensity (Supplemental Figure 12, E–G). Additionally, mitochondrial-specific markers DRP1, pDRP1, and OPA1 are enriched in KO cells while proteins of the oxidative phosphorylation pathway are not altered (Supplemental Figure 12, H and I). Thus, loss of KDM1A altered mitochondrial morphology and increased mitochondrial activity of nephron progenitor cells.

### CRISPR/Cas9-guided KO of KDM1A in human renal organoids recapitulates tubular cyst formation.

**Figure 6:**
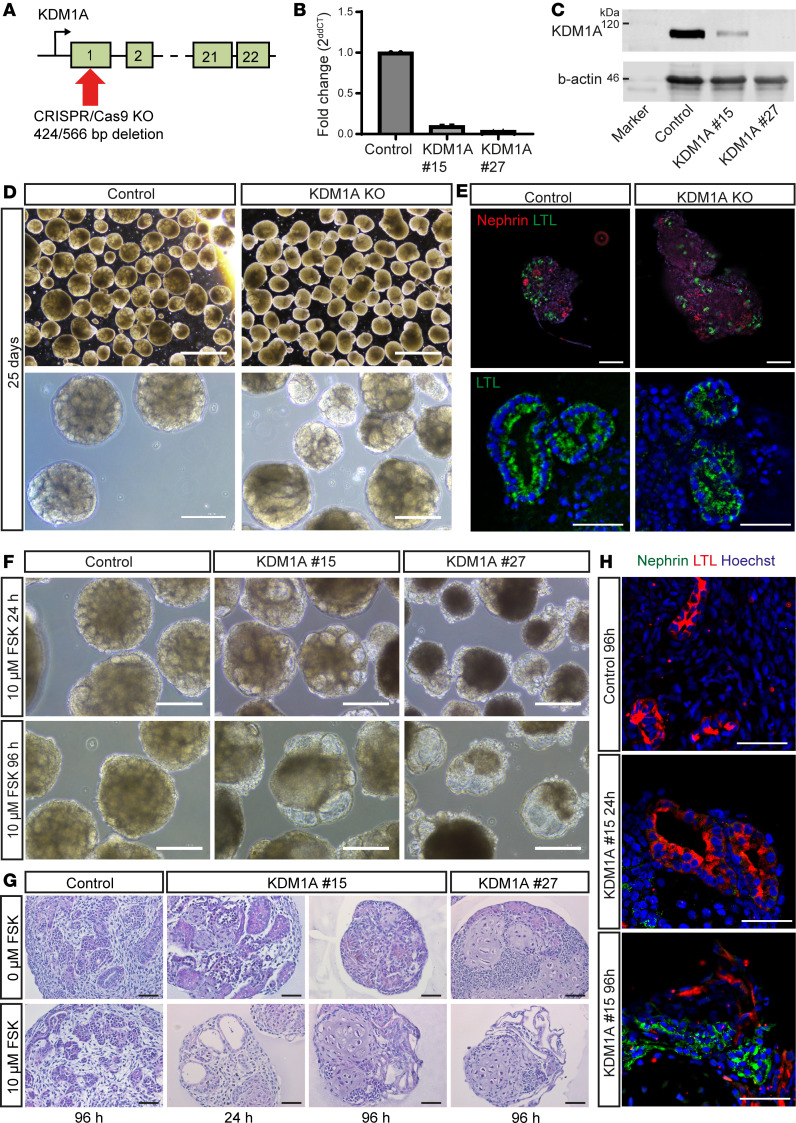
CRISPR/Cas9 LSD1 KO in human renal organoids leads to cyst formation. (**A**) CRISPR guide RNA was designed to target exon 1 of the *KDM1A* gene, analogous to the mouse KO, leading to a 424 bp (KDM1A #15) or 566 bp (KDM1A #27) frame shift deletion. (**B**) qPCR confirmed loss of *KDM1A* gene expression. (**C**) Western blot confirmed loss of KDM1A protein production. (**D**) Human kidney organoids after 25 days of differentiation show regular development and differentiation of KDM1A KO organoids. Scale bars: 1,000 μm (upper panel), 200 μm (lower panel). (**E**) IF staining of d25 control and KO organoids shows development of structures positive for podocyte marker Nephrin and proximal tubule marker LTL. Scale bars: 50 μm. (**F**) Organoids at day 32 after 24h of Forskolin treatment (upper panel) and day 35 after 96h of Forskolin treatment (10 μM, lower panel) show signs of cyst development in the KDM1A-KO organoids. Scale bars: 200 μm. (**G**) Histological sections of organoids at d32 and d35 show cysts after Forskolin treatment in the KDM1A-KO organoids. Scale bars: 100 μm. (**H**) IF staining of control and KO organoids at d32 or d35 Forskolin treatment (24h or 96h) shows development of cysts in structures positive for proximal tubule marker LTL. Scale bars: 100 μm.

Human kidney organoids have increasingly been used to study kidney development, kidney disease, and human mutations (35). In order to determine the role of human *KDM1A* for kidney development, we deleted a 424 bp DNA sequence in exon 1 in induced pluripotent stem cells (iPSCs; ERC001/UKEi001-A) using CRISPR/Cas9 technology (Figure 6A). Two iPSC clones, #15 and #27, with confirmed frameshift mutations were selected and screened for chromosomal abnormalities and off-target mutations. qPCR confirmed lack of *KDM1A* mRNA, and Western blot analysis showed reduction of KDM1A protein in both clones (Figure 6, B and C). The *KDM1A*-KO clones were subsequently used for differentiation into renal organoids (36, 37) (Figure 6D). Until d25, the organoids showed no differences compared with WT controls, with structures staining positive for proximal tubule marker LTL and podocyte marker Nephrin (Figure 6E). At this stage, no cyst formation could be detected (Figure 6E). To facilitate cyst formation, short-term treatment with Forskolin was applied, which activates adenylyl cyclase and leads to enhanced cAMP levels. In contrast to control organoids, the *KDM1A*-KO organoids showed rapid development of cysts and increasing cyst size within 24 and 96 hours of treatment, thereby replicating the murine cystic phenotype (Figure 6, F and G). Furthermore, the cysts can be localized to the tubular compartment by costaining with proximal tubule marker LTL (Figure 6H).

### snRNA-seq reveals upregulated metabolic pathways in KDM1A-KO organoids.

**Figure 7:**
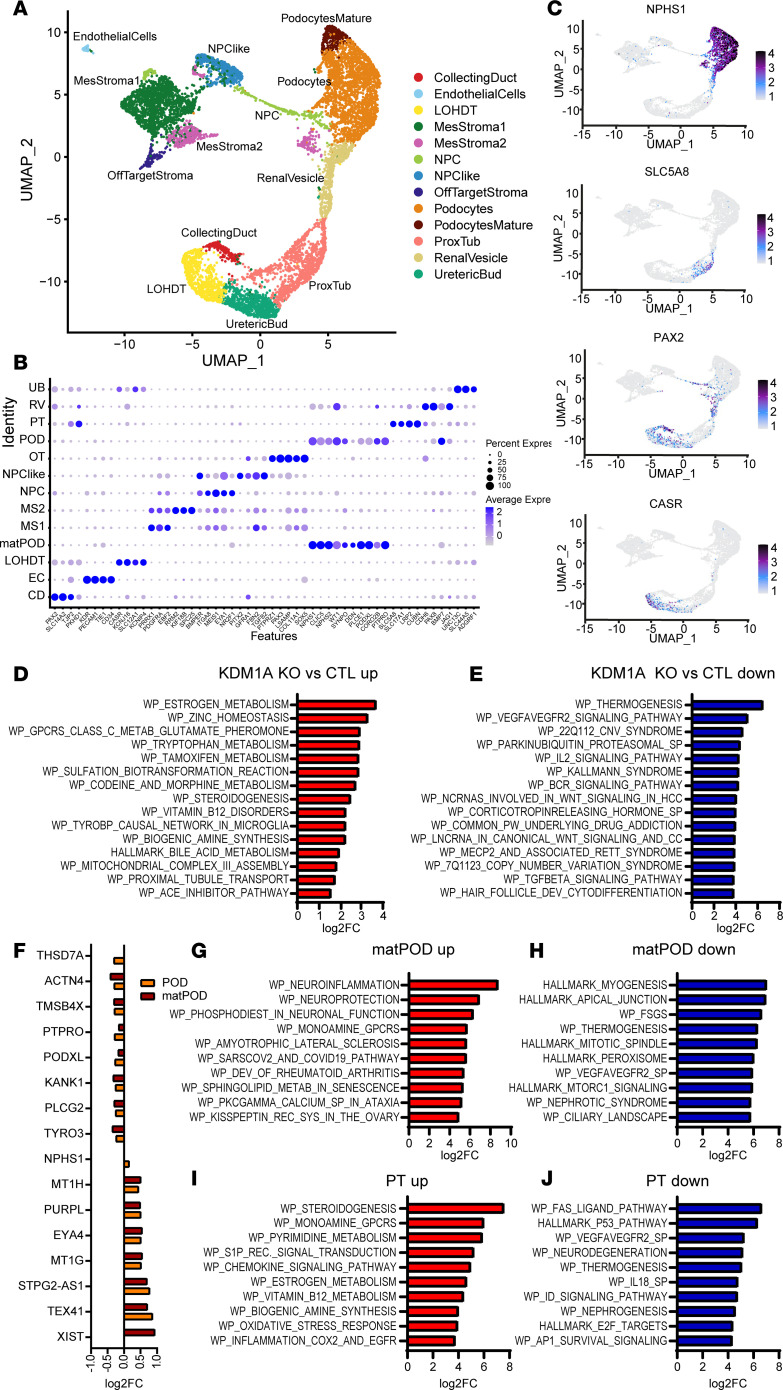
snRNA-seq shows differential gene expression and GSEA in the *KDM1A*-KO organoids. (**A**) UMAP of all sequenced cells. Annotated cell type is color-coded. (**B**) Marker gene expression per cell type. (**C**) Marker expression for podocytes (*NPHS1*), proximal tubules (*SLC5A8*), collecting duct (*PAX2*), and loop of Henle/distal tubules (*CASR*). Color code represents the scaled normalized intensity. (**D**) GSEA of upregulated genes across all cell types. (**E**) GSEA of downregulated genes across all cell types. (**F**) Bar chart of significantly up- or downregulated genes in podocyte subsets. (**G**) GSEA of upregulated genes in mature podocytes. (**H**) GSEA of downregulated genes in mature podocytes. (**I**) GSEA of upregulated genes in proximal tubule cells. (**J**) GSEA of downregulated genes in proximal tubule cells.

*KDM1A*-KO and control organoids at d25, before development of the cystic phenotype, were used for single nuclear RNA-seq. UMAP annotation shows distribution of cell types from mesenchymal cells to podocytes and tubule cells (Figure 7A). Cell type markers show distinct expression in the respective cell types, such as NPHS1 for podocytes, SLC5A8 for proximal tubules, PAX2 for collecting duct, and CASR for loop of Henle/distal tubules (Figure 7, B and C). Gene set enrichment analysis (GSEA) across all cell types shows upregulation of genes involved in metabolic functions, such as estrogen, tryptophan, and tamoxifen metabolism (Figure 7D). GSEA from downregulated genes indicates regulation of pathways such as thermogenesis, which has been implicated previously (31, 32), VEGFA/VEGFR2 signaling, Parkin/Ubiquitin proteasomal system, and IL-2 signaling (Figure 7E). Expression of genes in both podocyte and mature podocyte clusters show downregulation of genes such as *ACTN4*, *PTPRO*, *PODXL*, *KANK1*, and *PLCG2* (Figure 7F). This was confirmed by GSEA: While upregulated gene sets encompassed many inflammation-associated terms, such as neuroinflammation, SARS-CoV2, and rheumatoid arthritis (Figure 7G), downregulated gene sets include focal segmental glomerulosclerosis (FSGS) and nephrotic syndrome, both containing podocyte marker genes, as well as mTORC1 signaling, mitotic spindle, and ciliary landscape, indicating involvement in cell growth and proliferation (Figure 7H). In proximal tubules, GSEA of upregulated genes is dominated by metabolic gene sets, such as steroidogenesis, as well as chemokine signaling and oxidative stress response (Figure 7I). Downregulated gene sets in proximal tubules include p53 pathway, thermogenesis, and nephrogenesis (Figure 7J). Genes downregulated in both proximal tubules and loop of Henle/distal tubules include mitochondrial genes, *PLCG2*, *TMSB4X*, and *ARHGAP6* (Supplemental Figure 13). No consistent regulation of ciliary genes was detected, except for *DDX5* (Supplemental Figure 14).

### Upregulation of ncRNA in KDM1A-KO organoids is implicated in cell proliferation and cell growth.

**Figure 8:**
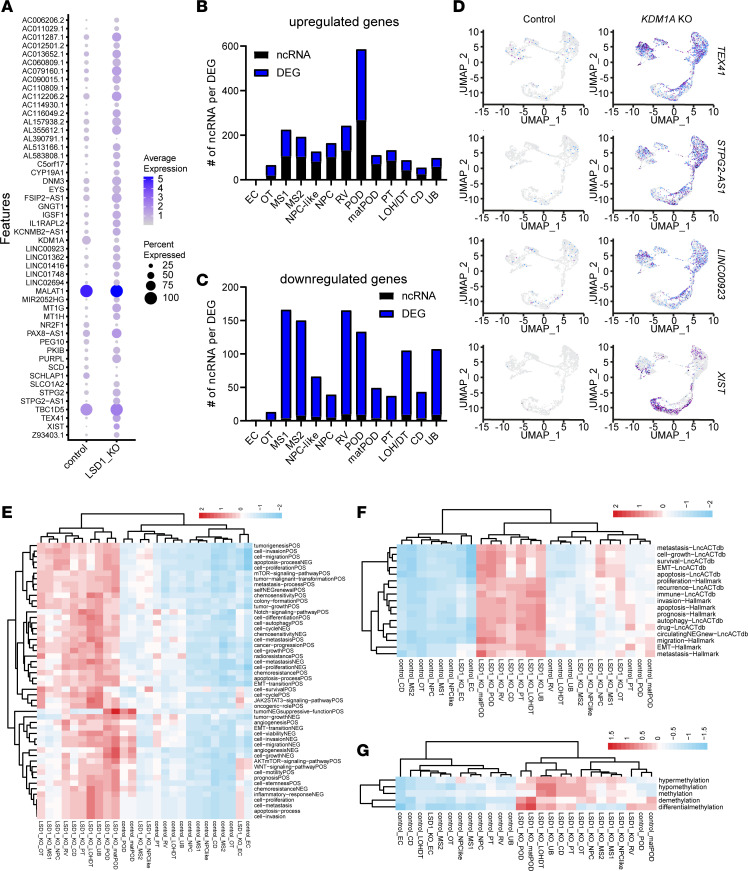
snRNA-seq shows upregulation of noncoding RNAs in the *KDM1A*-KO organoids. (**A**) Dot plot of the top 50 regulated genes across all cell types. (**B**) Proportion of noncoding RNAs of upregulated genes per cell type. (**C**) Proportion of noncoding RNAs of downregulated genes per cell type. (**D**) Expression of ncRNAs Tex41, STPG2 and XIST/LINC00923. (**E**) Average activity score (color coded) on gene sets “Experimental validated function” from lncSEA. (**F**) Average activity score on gene sets “Cancer functional state” from lncSEA. (**G**) Average activity score on gene sets “Methylation pattern” from lncSEA.

While some genes are differentially regulated only in individual cell types in the organoids, an overlap of differentially expressed genes across all cell types can be seen. The top 50 regulated genes across all cell types shows that the majority of these genes are upregulated. Furthermore, most of the genes (60%) can be attributed to the family of noncoding RNA (ncRNA), most of them long noncoding RNA (lncRNA) genes, such as *XIST*, *TEX41*, *PURPL*, *PAX8-AS1*, and *LINC00923* (Figure 8A). *KDM1A* appears as one of the top downregulated genes, again confirming the KO. When looking at the different cell types, ~53% of upregulated genes per cell type can be attributed to the ncRNA gene family (Figure 8B), while only ~7% of downregulated genes are ncRNA (Figure 8C and Supplemental Table 1). The top ncRNAs, such as *XIST*, *TEX41*, *STPG2-AS1*, and *LINC00923*, were upregulated in the majority of cell types (Figure 8D). Because the previously mentioned gene sets do not account for most ncRNA, we used lncSEA (38) for enrichment analysis. In the category “experimental validated function,” upregulation of lncRNA can almost exclusively be seen in the *KDM1A* KO organoid cell types, except for MS1, NPClike, and EC subtypes (Figure 8E). Among the enriched pathways are tumorigenesis, cell invasion, cell migration, cell proliferation, and mTOR signaling pathway. In the category “functional state,” the cell types of the maturing nephron cluster together and show enrichment of pathways such as metastasis, cell growth, and proliferation (Figure 8F). Furthermore, the category “methylation pattern” indicates enrichment of hypomethylation and hypermethylation, methylation and demethylation, and differential methylation, which is also present in control podocyte cell types (Figure 8G). In summary, prominent upregulation of ncRNAs, mostly lncRNAs, in the *KDM1A* KO organoids is associated with cell proliferation and cell growth.

## Discussion

Epigenetic regulation plays a pivotal role in renal development (15, 39–41) and disease (42, 43). However, the specific functions of many epigenetic modifiers in the context of the kidney remain elusive. In this study, our objective is to unravel the significance of the histone demethylase KDM1A in both renal development and function.

KDM1A has been shown to be indispensable for stem cell differentiation (18), and organ development, such as heart (44) by activation or decommissioning of histones, thereby regulating important transcriptional processes for cell differentiation. The present study shows that, in the kidney, *Kdm1a* is expressed during early nephron development in the nephrogenic zone, including CM and ureteric bud (UB) tips, as well as in the initial nephron stages of renal vesicles and comma-shaped bodies. However, mature nephrons and adult kidneys show less expression of *Kdm1a*. Thus, KDM1A only has a small window of time to “set the stage” for proper renal development and maintenance. At the time of birth, *Kdm1a*-KO animals show mild renal hypoplasia, indicating a disadvantage for renal growth and nephron number. The mild proteinuria at birth indicates a podocyte phenotype, which progressively worsens over time. The disease phenotype shows variability in disease severity, cyst numbers, and sizes, as well as survival of the *Kdm1a*-KO mice. Despite the substantial effect on podocytes and tubular cells in the adult mice upon early KO of *Kdm1a* in the nephron progenitor cells, conditional KO in the podocyte and tubular compartments did not manifest a phenotype. The onset of *Nphs2* expression coincides with the emergence of s-shaped bodies during glomerular development (45). Consequently, the deletion of *Kdm1a*, facilitated by Cre expression under the control of the *Nphs2* promoter, starts specifically at this developmental stage and depends on protein turnover. The same is true for *Kdm1a* deletion under the control of the *Pax8* promoter (46). Thus, *Kdm1a* deletion seems to take place after the crucial time window for KDM1A action in the podocyte and tubular compartments, and KDM1A-mediated histone demethylation seems to be dispensable for cell differentiation and function after the s-shaped body stage of the nephron. A similar phenomenon has been previously described, where inhibiting KDM1A during a short period in pancreatic endocrine cell development results in the failure of endocrine cell development. Conversely, inhibition at a slightly later stage of development leads to proper development (47). This suggests that the differentiation into mature cell types requires precise timing of KDM1A function for activating or repressing genes (48). Although KDM1A and KDM1B are paralogous demethylases, compensation by KDM1B appears unlikely due to the absence of upregulation in our datasets, and the relatively mild phenotype at birth may instead reflect persistence of KDM1A protein due to earlier gene expression and protein stability prior to Six2.Cre-mediated deletion.

Next to its role for histone demethylation, KDM1A has also been described as an interaction partner to a plethora of proteins, such as HDAC2 (23), CoRest complex (RCOR1, HDAC1, HDAC2, and others; ref. 24) DNMT1 (25), and others (26). Using a demethylase-deficient *Kdm1a*-KI mouse (31), we could confirm that the ablation of the KDM1A demethylase activity was sufficient to phenocopy the conditional deletion of KDM1A during nephron development. Thus, loss of the KDM1A demethylase activity seems to be the primary cause for nephron dysfunction. While the role of KDM1A for mitochondrial function has been described (32) and mitochondrial abnormalities were visible in TEM, no impairment of mitochondrial function could be measured.

Using human kidney organoids, we were able to recapitulate the cystic phenotype in the in vitro model system, thereby validating our findings in human tissue and indicating a species-overlapping function, leading to susceptibility for cyst development in the KO. snRNA-seq confirmed multiple features of *Kdm1a* KO, such as differences in metabolic function, including adaptive thermogenesis (32) and elevated mitochondrial activity (49), and metabolic pathways, such as lipid metabolism/glucocorticoid activation (32, 50). In the organoid podocytes, gene expression analysis revealed the downregulation of well-known causative genes for FSGS and nephrotic syndrome, such as *ACTN4* (51, 52), *PLCG2* (53), *KANK1* (54), *PODXL* (55), and *PTPRO* (56). The downregulation of individual or a combination of these genes can account for the impairment of podocyte function, proteinuria, and glomerulosclerosis and thus likely explains the renal *Kdm1a*-KO phenotype in the mice. Additionally, renoprotective genes *TYRO3* (57) and *TMSB4X* (58) were downregulated.

In the tubular structures, both mouse kidneys and human kidney organoids showed development of tubular cysts upon *KDM1A* KO, indicating an increased susceptibility to tubular dilation, increasing with age in the mouse or with Forskolin stimulation in the kidney organoids. Gene expression analysis in the proximal tubules indicate increased steroid metabolism, and inflammatory and oxidative stress response, as well as perturbed pathways connected to cell proliferation (downregulation of p53 pathway, E2F targets and AP1 signaling genes, tumor suppressor ARHGAP6; ref. 59). Furthermore, across most of the organoid cell types, ncRNAs were upregulated, while only few ncRNAs were downregulated. Most of the ncRNAs can be classified as lncRNAs (>200 nt, polyadenylated) and contain well-characterized members such as *XIST* and *PURPL* (60), or antisense transcript, such as *PAX8-AS1*, *STPG2-AS1*, or *SEMA6A-AS1*. While previous published data sets have not described a global regulation of lncRNAs in *KDM1A* KO or inhibition yet, KDM1A has been described to physically interact with lncRNAs, such as *HOTAIR* (61), *AGAP2-AS1* (62), or *HOXA11-AS* (63), leading to regulation of other genes. However, recently, the role of KDM1A for enhancer decommissioning and activation or repression of enhancer RNAs (eRNAs) has come more and more in the focus (64). As eRNAs are smaller (<2,000 nt) and mostly nonpolyadenylated, most of them would not have been picked up in our snRNA-seq analysis. However, one of the most upregulated transcripts in all cell types, *TEX41*, has recently been categorized as an eRNA (65) and was implicated in malignant cell growth. Furthermore, upregulated lncRNAs *PURPL* (66), *XIST* (67), and *MIR2052HG* (68) have also been linked to cancer and promotion of cell proliferation. *KDM1A* deletion has been implicated in enhancer derepression by promoting the binding of KLFs to KDM1A-targeted enhancers and recruitment of P300 to derepress KDM1A target genes (26). The ectopic transcription due to inappropriately inherited histone methylation has been described previously to interfere with the ongoing function of terminally differentiated cells (69). Moreover, the depletion of KDM1A has been demonstrated to destabilize DNMT1, resulting in gene derepression via DNA hypomethylation (25). Thus, our data suggest KDM1A-associated ectopic transcription of ncRNAs, including lncRNAs, as a cause for the increased susceptibility of tubules for cell proliferation and cyst growth in the *KDM1A*-deficient nephron.

In summary, our study highlights the requirement for KDM1A-mediated histone demethylation for proper nephron development and long-term renal function during a critical time window of nephrogenesis. Failure of KDM1A function in early stages of nephron development in the mouse results in long-term dysregulation of podocyte function and tubular cyst formation, a phenotype that can also be reproduced in human kidney organoids. snRNA-seq indicates altered metabolic processes, downregulation of podocyte genes associated with disease pathogenesis, and upregulation of ncRNAs (lncRNA, eRNA) altering long-term renal programming and susceptibility to cyst formation.

## Methods

Supplemental Methods are available online with this article.

### Sex as a biological variable

Male and female mice were are used for analysis, and similar findings are reported for both sexes.

### Animals

The strategies for conditional deletion of the first exon of *Kdm1a* (Lsd1^tm1Schüle^; hereafter *Kdm1a^fl/fl^*) (28) and conditional KI of an enzymatically inactive Kdm1a- variant (C57BL/6-Kdm1a^tm2931(K662A, W752A, Y762S)Arte^; hereafter *Kdm1aKI/KI*) (31) were described previously. The targeted ablation in nephron progenitor cells was generated using Six2-TGC^tg Tg(Six2-EGFP/cre)1Amc/J^ (hereafter Six2Cre) (29) purchased from Jackson Laboratory (Bar Harbor, ME, USA). The reporter mouse strain Gt(ROSA)26Sor^tm4(ACTB-tdTomato,-EGFP)Luo^/J (hereafter *tomato^fl/fl^*) (30) purchased from Jackson Laboratory enabled linage tracing of KDM1A-deficient cells by membranous GFP expression. hNPHS2Cre mice were used for podocyte-specific deletion of *Kdm1a* and a gift of Lawrence Holzman (Renal, Electrolyte, and Hypertension Division, University of Pennsylvania School of Medicine, Philadelphia, PA) (70). *Pax8*rtTA mice were from Robert Koesters, University Hospital Heidelberg, Heidelber Germany (46) and *tetO* mice were from Susan Quaggin, University of Toronto, Toronto, Canada (71). Cre- system was used for targeted ablation in tubules cells. Fourteen days of doxycycline treatment started at E8.5 via oral doxycycline administration of the pregnant female.

Breeding and genotyping were performed according to standard procedures. Tail biopsies were prefrozen at –20°C, then lysed at 95°C in an alkaline lysis reagent pH 12 (25 mM NaOH, 0.2 mM Na2EDTA) and neutralized with 40 mM TrisHCl pH 5. Primer sequences are listed in Supplemental Table 2.

### In situ hybridization

Generation of probes and the in-situ hybridization were performed as described previously (72). The primer sequences for *Kdm1a* and *Six2* have been found on the GenitoUrinary Development Molecular Anatomy Project (GUDMAP) gene expression database and were modified after Chad Vezina, University of Wisconsin-Madison, Madison, WI (*Kdm1a*) and Melissa Little, Murdoch Children’s Research Institute, Melbourne (*Six2*). Whole mRNA from E19.5 mouse kidney (WT) served as a template for RT-PCR (QIAgen OneStep RT-PCR Kit). Primer sequences are listed in Supplemental Table 2. Probes were labeled with alkaline phosphatase-conjugated anti-digoxigenin antibody (Roche Applied Science). In situ hybridization was performed on 10 μm thin sections at E14.5 mouse embryo, p0, and p42 kidneys according to standard procedure. The analysis was done using an Axioplan 2 microscope (Zeiss).

### Western blot analysis

Lysation of samples was performed in either tissue lysis buffer (RIPA, β-mercaptoethanol, Na3VO4, Roche Ultra complete proteinase inhibitor cocktail, Roche phosphostop) or cell lysis buffer (0,1% NP40, 1xPBS, Na3VO4, Roche tablet). Cell lysates were additionally sonicated. For protein quantification Thermo Scientific Pierce BCA Protein Assay was used according to manufacturer’s instructions. Lysed samples were diluted with 2x laemmli sample buffer with SDS and DTT and boiled at 42°C for 30 min. Western blot analysis was performed according to standard procedures (73). Protein transfer was performed using Trans-Blot Turbo Transfer System by BioRad (1.3 A and 25 V for 7 min). BSA (5%) in 1x wash buffer was used for blocking (1h at 37°C) as well as for dilution of primary and HRP-linked secondary antibodies. All used antibodies and the dilutions are listed in Supplemental Table 3.

### Histological and immunofluorescence analysis

Kidneys were fixed in 4% Paraformaldehyd (PFA, Sigma-Aldrich), dehydrated using a histocinette (Leica) and embedded in paraffin. For histology, sections of 3 μm slices were used for periodic acid–Schiff (PAS) reaction and acid fuchsin-orange G (SFOG) staining for histological analysis. SFOG and PAS staining procedures were performed in the Department of Pathology, University Hospital of Freiburg, Germany. Images of kidneys were acquired using an Axioplan 2 microscope (Zeiss) equipped with 10x, 20x, and 63x objectives and an AxioCam camera (Zeiss).

For IF of sections, 3 μm slices were deparaffinized in histoclear and rehydrated. Heat-induced epitope retrieval (HIER) was then performed in TRIS/EDTA pH9 for 30 min using a food steamer. BSA (5%) in 1xPBS was used for blocking (at RT) as well as for dilution of primary and secondary antibodies. Antibody incubation (at RT) took 1h with primary and 30 min with fluorophore-conjugated Alexa secondary antibodies (Invitrogen). Sections were carefully covered with Prolong Gold Antifade (Invitrogen) and the analysis was done using an Axio Imager.M2 immunofluorescence microscope equipped with 20x, 63x, and 100x objectives, with an apotome ApoTome.2 device, HXP lamp, fluorescence filter sets and an AxioCam503 camera (Zeiss).

For IF of primary cells, 7,000 cells/well were seeded into CollagenIV coated 8-well plate (μ-slide 8-well, ibidi). Attached cells were fixed in 4% PFA as well as permeabilized using 0,2% Triton X100 in 1xPBS. Staining was performed according to standard protocol. The analysis was done using an Axio Obserer.Z1 immunofluorescence microscope equipped with 20x and 63x objectives, with an ApoTome.2 device, Colibri.2 lamp, fluorescence filter sets and an AxioCam503 camera with an apotome (Zeiss). All used antibodies and the dilutions are listed in Supplemental Table 3.

### TEM

Dissected kidneys were carved transversally and fixed in 4% PFA with 1% Glutaraldehyde (Carl Roth) for 24 hours. Samples were postfixed in 1% osmium tetroxide in the same buffer for 1 hour and stained en bloc in 1% uranyl acetate in 70% ethanol for 1 hour, dehydrated in ethanol, and embedded in Durcopan (Plano, Wetzlar, Germany). Thin sections were stained with lead citrate and examined in a Zeiss Leo-906 TEM.

### Scanning electron microscopy

Dissected kidneys were cut into slices and examined using scanning electron microscopy. Samples were fixed overnight at 4°C in 0.1M cacodylate buffer containing 4% paraformaldehyde and 2% glutaraldehyde. Samples underwent dehydration through an ascending ethanol (EtOH) series (70%, 80%, 90%, 100%), with 15-minute incubations at each concentration. Samples were then treated with a 1:1 mixture of EtOH and hexamethyldisilazane (HMDS), followed by 100% HMDS, and allowed to air-dry. All specimens were sputter-coated with platinum using a Leica EM ACE600. Imaging was conducted on a Thermo Scientific Quattro ESEM electron microscope.

### Nephron count

Kidneys of *Six2.*Cre^+^ *mTomato/mEGFP^fl/fl^ Kdm1a^fl/fl^* or *Six2*Cre^+^ *mTomato/mEGFP^fl/fl^ Kdm1a^+/fl^* mice were dissected at p0 and fixed in 4% PFA. The organs were then cleared according to the X-CLARITY Tissue Clearing System protocol provided by Biozym scientific. Cleared kidneys were placed in 35 mm imaging dishes with glass bottom filled with X-CLARITY Mounting Solution and fixed with round cover glass (Ø24 mm). Z-stacks were then taken using an LSM 880 Observer.Z1/Fast Airyscan with inverted microscope (Zeiss) and a 10x objective. ImageJ software 1.50g was used to count all (pre-)mature glomeruli for determination of nephron number (multipoint tool) and for calculating the volume of the Z-stacks (Voxel Count).

### Renal physiological analysis

Urinary albumin-to-creatinine ratio (in mg/L) was determined at day of birth (p0) and once per week subsequently using the Creatinine PAP Kit by LT-SYS Labor+Technik (Eberhard Lehmann GmbH) and the Mouse Albumin ELISA Quantification Kit by Bethyl Laboratories according to manufacturer’s instructions. Concentration of urea (in mg/dL) in blood serum was measured at day of birth (p0) or at adulthood using the Urea Kit by LT-SYS Labor+Technik (Eberhard Lehmann GmbH) according to manufacturer’s instructions.

### Isolation of progenitor/primary cells

Kidneys were dissected at E14.5 (RNA-Seq) and p0 (cell culture), precut into small pieces and digested for up to 30 min shaking at 1,400 rpm and 37°C (0.75 mg Pronase, 1.065 mg 310U CollagenaseII, 5.33 μL 100U DNaseI in 1 mL 1xHBSS). For mechanical support of the cell isolation, the samples were sheered with a 27G needle. The expression of eGFP-Cre fusion protein of Six2Cre positive mice enabled specific isolation of nephron progenitor cells by fluorescence-activated cell sorting (BD FACSAriaIII). For primary cell culture, after 10 min of digestion the cellular suspension was sieved through cell strainer (100 μm and 70 μm, Corning). Isolated cells were cultivated with primary culture medium (DMEM7F12, Hepes, Fortecortin, EGF, L- Thryox, Penicillin-Streptomycin, ITS, FCS) at 37°C.

### Mitochondrial analysis

The metabolic function of the mitochondria was analyzed using the Seahorse XFp mitochondrial stress test (Seahorse Bioscience Billerica, MA). Mitochondrial membrane potential was analyzed using TMRM fluorescence (T668, Thermo Fisher Scientific) and MitoTracker (M7514, Thermo Fisher Scientific) costaining of living cells isolated from *KDM1A* KO and WT littermates. In total, 15,000 cells were cultured on fibronectin (#354008, Corning) precoated Ibidi μ-dish overnight and stained with 100 nM TMRM and 100 nM MitoTracker for 30 minutes. A TOM20 antibody (sc-11415) was used for visualization and analysis of mitochondria by standard immunofluorescence as described above. Fluorescence intensities and cell areas were measured using Fiji ImageJ v1.51. For TMRM signal analysis, a selection mask of mitochondria covered areas was created using the MitoTraker signal and TMRM to MitoTraker signal ratio was measured.

CM-H2DCFDA General Oxidative Stress Indicator (C6827, Thermo Fisher Scientific) was used for ROS measurement on a 96-well plate (Nunc 96-Well Microplates for Fluorescence-based Assays). 15000 cells per well were seeded on a fibronectin precoated 96-well plate, cultured overnight and stained with 50 μM CM-H2DCFDA in 1xHBSS for 30 min. After carefully washing in HBSS fluorescence intensities were measured using a plate reader (Tecan). All assays were performed according to manufacturer’s instruction.

### CRISPR/Cas9 KO

#### Design guide RNA.

Identification of suitable PAM sequences and design of the 20-nt long sgRNAs flanking the deletion site (gRNA Exon1_start: GGCAAGGCTTTTCGGACCCA-CGG;

gRNA Exon1_end: GGCGGTGTCGTTTGAGGGAA-GGG) was done with the online CRISPR design web tool CRISPOR (74). Individual gRNAs (Alt-R CRISPR-Cas9 crRNA) were produced at Integrated DNA Technologies (IDT).

#### HiPSC culture.

The human iPSC cell line UKEi001-A (https://hpscreg.eu/cell-line/UKEi001-A, cellosaurus ID nr: CVCL_A8PR) was used as the parental cell line and cultured in mTeSR (Stemcell Technologies) on Matrigel (Corning, 1:60) coated plates.

#### Nucleofection and clone selection.

One hour before nucleofection, hiPSCs were incubated with Y-27632 (Biorbyt, 10 μM). The CRISPR/Cas ribonucleoprotein (RNP) complex was assembled by first annealing equal amounts of site-specific Alt-R CRISPR-Cas9 crRNA with universal Alt-R tracrRNA-ATTO^550^ (IDT, 5 min, 95 °C, final concentration 50 μM) and then combining with Alt-R S.p. Cas9 Nuclease (IDT) at a ratio of 1:1.5 (60 min, RT). HiPSCs were dissociated with Accutase (Sigma, 5 min, 37°C) into single cells. For nucleofection, 0.8x10^6^ hiPSCs were resuspended in 100 μL “P3 solution” (Lonza P3 Primary Cell 4D-Nucleofector X Kit, prepared by mixing 82 μL “Nucleofector solution” with 18 μL supplement) according to the manufacturer’s instructions. Finally, the CRISPR/Cas RNP and 1 μL electroporation enhancer (IDT, 100 μM) were mixed with the 100 μL cell suspension and transferred to the Amaxa nucleofection cuvette. Nucleofection was performed with the Amaxa 4D-Nucleofector (Lonza) according to the manufacturer’s instructions. Nucleofection program CA-137 was applied. After nucleofection, the cells were incubated for 5 min in the cuvette at 37°C. HiPSCs were plated on Matrigel-coated 12-well culture dishes (0.8 × 10^6^ hiPSC per well) in mTesR supplemented with CloneR (Stemcell Technologies, 1:10). Medium was changed after 24 h. After 48 h, nucleofected hiPSCs were dissociated with Accutase (5 min, 37°C), and plated at a density of 3.0 × 10^3^ cells per Matrigel-coated 6-well in mTeSR supplemented with CloneR. After 10–14 days of hiPSC expansion, individual clones were picked (10 μM Y-27632 1 h prior to picking) and transferred to 48- and subsequently 24-well Matrigel-coated dishes. Copy plates in the 24-well format were generated, and aliquots were frozen. DNA was isolated and sequenced to test the genome editing efficiency as well as to exclude modification of the 10 most probable off-target loci (Supplemental Table 4). Suitable clones were chosen and sent for karyotyping (StemGenomics, iCS-digital PSC 24-probe test). For determination of pluripotency by flow cytometry, anti-SSEA-3 antibody (PE-Anti-SSEA3 1:5 dilution [BD Biosciences #7167881]) was used and compared with the corresponding isotype control (PE-Rat IgM, κ Isotype Control 1:40 dilution [BD Biosciences #7152801]) and compared with using a FACSCanto II Flow Cytometer (BD Biosciences).

### Kidney organoid differentiation

Kidney organoids were generated following a modified Takasato protocol (75, 76). HiPSCs were dissociated into single cells using Accutase (Sigma, 5 min, 37°C), seeded onto Matrigel-coated (Corning, 1:60) 6-well plates (Nunc) at a density of 12,000 cells/cm^2^ in Essential 8 medium (E8, Thermo Fisher Scientific) with Y-27632 (10 μM, Biorbyt), and incubated overnight at 37°C and 5% CO_2_. This hiPSC monolayer was cultured in Essential 6 medium (E6, Thermo Fisher Scientific) supplemented with 7 μM CHIR99021 (Sigma) from day 1 to day 4, followed by 200 ng/mL FGF9 (Peprotech), 1 μg/mL heparin (Stemcell Technologies) and 1 μM CHIR99021 from days 5 to 7. To form organoids, cells were then dissociated using Trypsin (Gibco), washed with E6 medium and centrifuged at 200g. The cell pellet was resuspended in Stage 1 medium [E6 medium containing 200 ng/mL FGF9, 1 μg/mL heparin, 1 μM CHIR99021, 0.1% polyvinylacohol (PVA, Sigma), 0.1% methylcellulose (MC, Sigma), 10 μM Y-27632] and transferred to 6-well plates pretreated with Pluronic-F12 (Sigma) for low adhesion conditions (day 7+0). Cell aggregates spontaneously formed by rotating the culture dishes on an orbital shaker (Thermo Fisher Scientific) at 70 rpm incubated at 37°C and 5% CO_2_. After 24 h medium was switched to Stage 2 medium [E6 medium containing 200 ng/mL FGF9, 1 μg/mL heparin, 1 μM CHIR99021, 0.1% PVA, 0.1% MC) for another 4 days (dd7+1 to d7+4). From day 7+5 onward, organoids were cultured in Stage 3 medium (E6 medium containing 0.1% PVA, 0.1% MC) and kept on the orbital shaker until end of experiment. Organoid cultures (whole wells of cells, isolated spheroids and/or overlying culture medium) were collected at indicated time points from seeding.

### snRNA-seq

Organoid cultures were collected on day 24 in 500 μL RNAlater and immediately frozen at –80°C. For single nucleus dissociation, the organoids were thawed and washed with additional 1 mL DPBS (59331C; Sigma) at 1,000*g* for 5 min at 4°C. The pellet was dissociated and homogenized using a Dounce homogenizer (D8938-1 SET, Sigma-Aldrich) in 200 μL ice-cold lysis solution and incubated on ice for 20-30 min with additional 3.8 mL of ice-cold lysis solution. Lysis solution was prepared with Nuclei PURE lysis buffer (NUC-201, Sigma-Aldrich), 1 mM dithiothreitol (D9779, Sigma-Aldrich) and 0.1% Triton X-100 (NUC-201, Sigma-Aldrich) according to manufacturer protocol and a RNAse inhibitor mix (0.04 U/μL SUPERaseIN RNAse Inhibitor [AM 2696, Thermo Fisher]; 0.04 U/μL RNAsin Plus RNAse Inhibitor [N2615, Promega]) was added. The single nuclei suspension was filtered through a 30 μm strainer (04-004-2326, Sysmex) and centrifuged at 500g for 5 min at 4°C. The pellet was resuspended in 1 mL of ice cold 0,01% BSA (AM2616, Thermo Fisher) in DPBS (59331C; Sigma) with 0.04 U/μL SUPERaseIN RNAse Inhibitor and 0.04 U/μL RNAsin Plus RNAse Inhibitor, filtered through a 5 μm strainer (04-004-2323, Sysmex) and washed with additional 4 mL of ice cold 0,01% BSA at 500g for 5 min at 4°C. The pellet was resuspended in 2% BSA in DPBS with 0.04 U/μL SUPERaseIN RNAse Inhibitor and 0.04 U/μL RNAsin Plus RNAse Inhibitor and nuclei were counted.

The libraries were prepared with the Chromium NEXT GEM Single Cell 3′ Reagent kits v3.1 according to manufacturer’s protocol with a capture rate of ~ 10,000 nuclei. The libraries were sequenced on an Illumina Novaseq6000 platform as symmetric paired end runs (150 bases) with 200 million raw sequencing reads per sample. Organoid snRNA-seq paired fastq files were processed with 10x Genomics Cell Ranger 6.1.2 (77) using Human reference genome GRCh38 (2020-A) downloaded from the 10x Genomics. Downstream analysis was performed using Seurat 4.3.0.1 (78). The 3 samples were integrated into a single dataset using sctransform V2 from Seurat. The following thresholds were used to remove low quality content: number of quantified genes per cells between 200 and 5000, number of cells expressing a gene > 3, percentage of mitochondrial content < 10. A total of 9180 cells were filtered-in. Principal Component (PC) Analysis was computed on the top 5000 most variable genes. Based on the PC elbow plot, the top 20 PCs were used to calculate the shared Nearest-neighbor graph. Subsequently, Louvain clustering was executed with a resolution of 0.3, and the resulting clusters were rendered via Uniform Manifold Approximation and Projection (UMAP). Putative cell types were discerned utilizing the sctype R package. Marker genes were identified employing a nonparametric Wilcoxon test, with significance determined by an adjusted *P* value threshold of less than 0.05. Marker genes were used to perform GSEA using Fisher’s exact test with the MSigDB as reference gene-sets. For lncSEA, the gene-set score was calculated per cell using Decoupler R package with the Univariate Linear Model method (79).

### Experimental analysis and statistics

If not mentioned otherwise, all data represent the mean values ± SEM under consideration of SD and number of measurements (*n*). Independent biological replicates are stated as *N*. Statistical significance between 2 groups was assessed using an unpaired 2-tailed Student’s *t* test. For experiments involving more than 2 groups, statistical analysis was performed using 1-way ANOVA followed by Dunnett’s multiple-comparison correction. Statistical significance is indicated by asterisks. All analyses were performed using GraphPad Prism.

### Study approval

All mouse experiments were performed according to the approval by the committee on Research Animal Care of the Regional Council in Freiburg (authorization nos. G-11/51 and G-16/148). All mice were housed in the Center for Experimental Models and Transgenic Service (CEMT) of the University Hospital Freiburg under specific pathogen-free (SPF) conditions and with a 12-h day/night cycle, free access to water and standard rodent chow.

All procedures involving human cells were approved under study 2021-10572_4-BO-ff (*hiPSC-Modelle für die biomedizinische Forschung*). Informed consent was obtained from all donors prior to sample collection.

### Data availability

The data are available at https://www.fdr.uni-hamburg.de/record/14226, DOI 10.25592/uhhfdm.14226, and in the Supporting Data Values file. Raw sequencing data from human cells are available from the corresponding author upon reasonable request, subject to approval by the relevant ethics committee and in accordance with data protection laws.

## Author contributions

Conceptualization was contributed by NW, JK, NL, WBW, and TBH. Investigation was contributed by JK, NL, SDL, SEG, MR, WL, TB, CM, MH, OK, IH, NW, FB, FH, MNW, VGP, and EM. Data curation was contributed by JK, NL, MR, MB, and GA. Writing of the original draft was contributed by JK and NW. Review and editing were contributed by NW, JK, NL, MR, RS, WBW, and TBH. Funding acquisition was contributed by NW, WBW, and TBH. Supervision was contributed by NW, WBW, and TBH. NW, JK, and NL contributed equally to this work. The order of co–first authors was determined by mutual agreement based on their comparable overall contributions to experimental design, data acquisition, and manuscript preparation.

## Funding support

- Collaborative Research Centers CRC1192 (Project ID 264599542 to TBH, VGP, and NW), CRC1160 (Project ID 256073931-Z02, to MB), CRC/TRR167 (Project ID 259373024-Z01, to MB), CRC1453 (Project ID 431984000-S1, to MB), CRC1479 (Project ID 441891347-S1, to MB), TRR359 (Project ID 491676693-Z01, to MB), TRR 353 (Project ID 471011418-SP02 to MB), CRC1648 (Project ID 512741711 to TBH), CRC1453 (Project ID 431984000 to TBH), TRR422 (Project ID 543604419, to NW, FB, VGP, and TBH), and FOR5476 UcarE (Project ID 493802833-P7, to MB)
- HU 1016/8-2, HU 1016/11-1, HU 1016/12-1 (to TBH), Schu688/15-1 (to RS), and BR 6668/2-1 to FB
- SFB 992 Medical Epigenetics (Project ID 192904750, to RS and TBH)
- Federal Ministry of Education and Research (BMBF) projects STOP-FSGS (FKZ 01GM2202A, to TBH, VGP, and NW), PM4Onco (FKZ 01ZZ2322A, to MB), Fibromap (FKZ 01ZX1914A, to VGP), and EkoEstMed (FKZ 01ZZ2015, to GA)
- Federal Ministry of Research, Technology and Space (Funding No. 01EO2106; to FB)
- Else-Kröner Fresenius Foundation Else Kröner-Program – iPRIME (to TBH, scholarship to FH)
- Grant 2016_Kolleg.03 (to WBW and MR)
- ERC advanced grant (CureFSGS, 101141768 to TBH)
- Marie Curie EU Grant (CIG 293568; to WBW)
- Margarete von Wrangell Habilitation Program (Ministry of Science, Baden-Württemberg; to WBW)
- Mathilde-Wagner-Habilitationspreis (to WBW)
- “Fill in the Gap” scholarship of the Faculty of Medicine, Albert-Ludwigs-University Freiburg (Germany; to JK)
- Deutsches Konsortium für Translationale Krebsforschung (DKTK) grant DKTK FR01-374 (to EM)

## Supplementary Material

Supplemental data

Unedited blot and gel images

Supplemental table 1

Supporting data values

## Version 1 03/09/2026

Electronic publication
