# Supplementary material for: Lysine-specific histone demethylase 1a regulates nephron development and long-term transcriptional programming
Source: JCI Insight. 2026 Mar 9;11(5):e190283. doi: 10.1172/jci.insight.190283 (PMC13041676; doi:10.1172/jci.insight.190283)
Supplement: Supplemental data [file jciinsight-11-190283-s233.pdf]

Supplementary Material

Supplementary Methods:

Bulk RNA-seq

Six2.Cre-eGFP-positive cells were isolated from five heterozygous and five knockout (KO) mice at E14.5 after enzymatic digestion. Single cells were FACS-sorted using a BD FACSAriaTMIII cell sorter. RNA was extracted using the phenol/chloroform-method and sequenced using the Illumina HiSeq2000 platform with 125-bp paired-end reads. Low-quality reads were identified using FastQC and removed with Trimmomatic[1]. High-quality reads were aligned to the mouse reference genome (mm9) using the STAR aligner[2], and gene-level counts were quantified. Differential gene expression between KO and heterozygous samples was analyzed using the DESeq2 pipeline[3]. Genes with an adjusted *P*-value (Benjamini–Hochberg correction) below 0.05 were considered significantly differentially expressed.

Supplementary Figures:

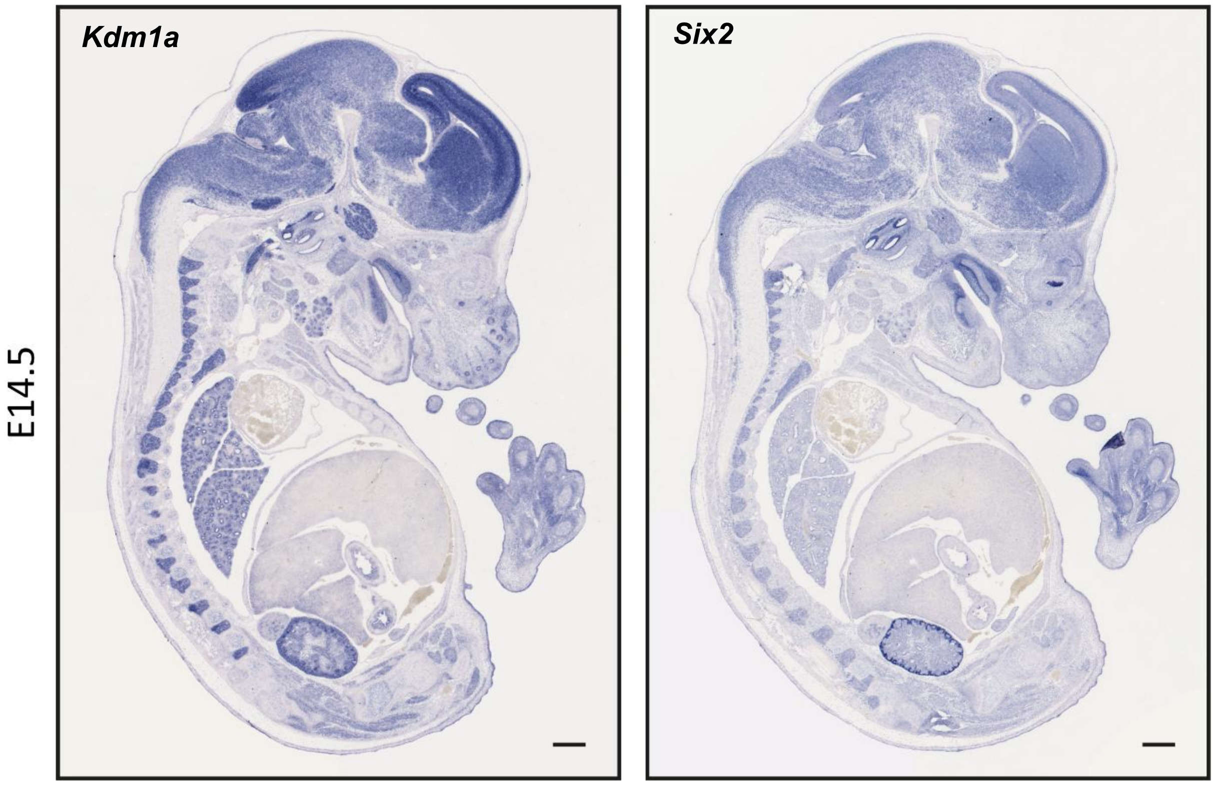

Supplementary Figure 1. In situ hybridization for *Kdm1a* and *Six2* in E14.5 kidneys. In situ hybridization on embryonic day 14.5 (E14.5) wild-type kidney sections detecting *Kdm1a* (left) and *Six2* (right) mRNA expression. Scale bar: 1 mm.

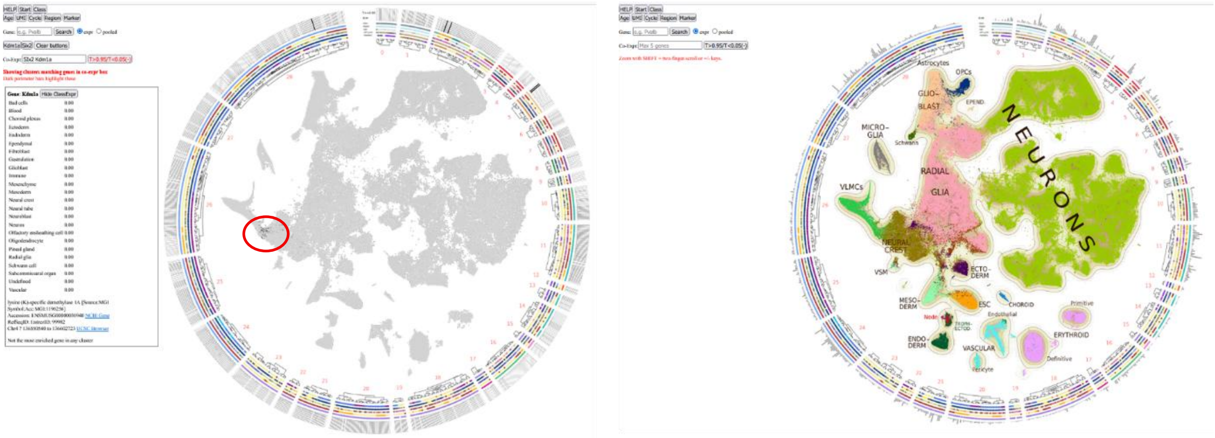

Supplementary Figure 2. Overlapping expression of *Six2* and *Kdm1a* in the developing mouse brain. At embryonic day 10.5 (E10.5), overlapping expression of *Six2* and *Kdm1a* in the developing mouse brain is restricted to neural crest–derived cells and vascular/leptomeningeal cells (VLMCs), based on data from the Mouse Brain Atlas (http://mousebrain.org/wheel/)[4].

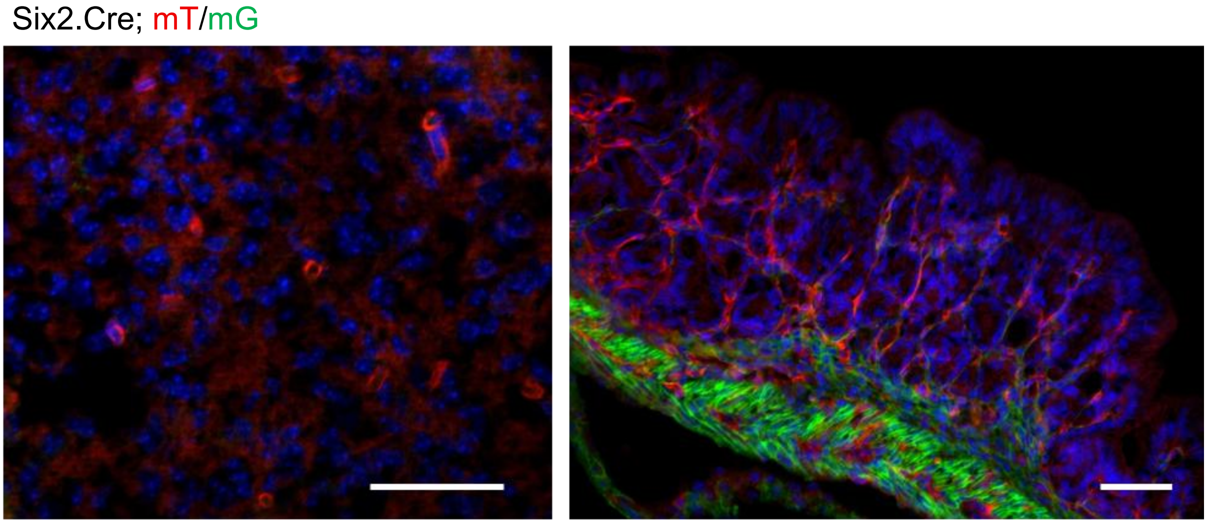

Supplementary Figure3. Six2.Cre-driven reporter expression in non-renal tissues. Tissue sections from postnatal day 0 (p0) Six2.Cre;mTomato/mEGFP reporter mice stained with DAPI. Cre activity is indicated by mEGFP expression (green), while mTomato (red) marks non-recombined cells. No Cre activity is detected in the cerebral cortex (left), whereas mEGFP-positive cells are observed in the stomach (right). Scale bar: 50 µm.

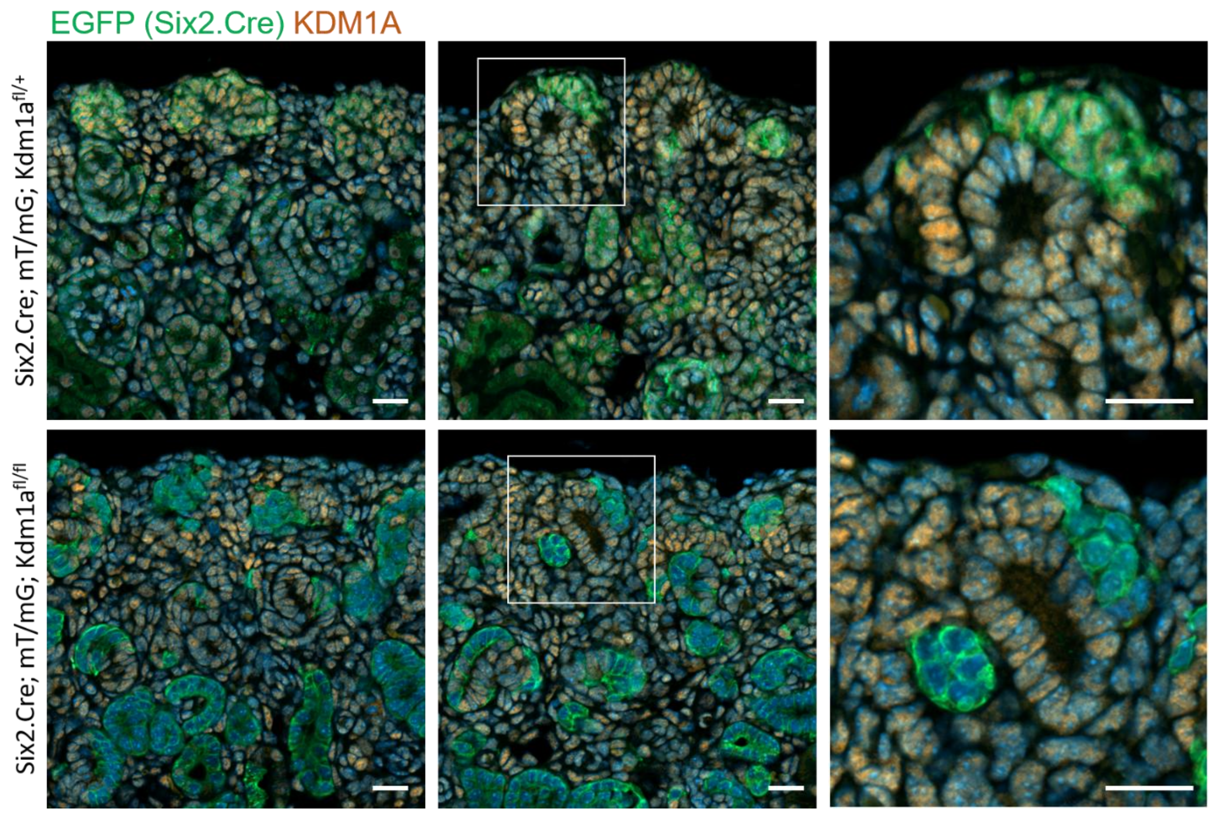

Supplementary Figure 4. Validation of *Kdm1a* knockout in p0 kidneys. Immunofluorescence staining for KDM1A (orange) and EGFP (green) in postnatal day 0 (p0) kidneys from heterozygous (upper panel) and knockout (lower panel) mice. In knockout kidneys, KDM1A staining is absent in EGFP-positive cells, confirming successful gene deletion. Scale bars: 20 µm.

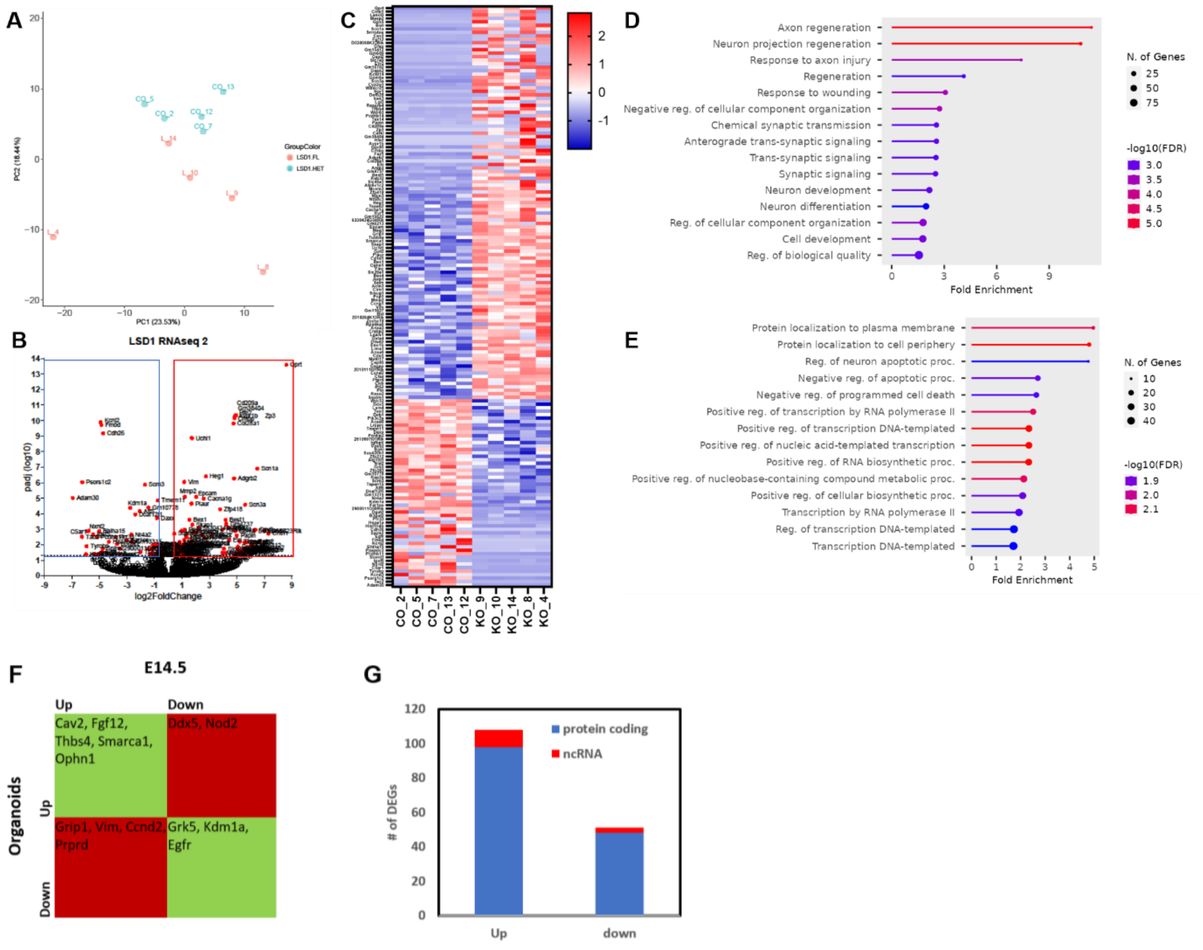

Supplementary Figure 5. RNA-sequencing analysis of isolated cap mesenchyme at E14.5. A) Principal component analysis shows clear differentiation between control (Six2.Cre;Kdm1a$^{fl/+}$ (heterozygous, LSD1_HET)) and KO (Six2.Cre Kdm1a$^{fl/fl}$ (LSD1_KO)) cells. B) Vulcano shows distribution of differentially regulated genes. C) Heatmap of differentially regulated genes per sample. D and E) Shiny GO plot of top 15 GO Biological Processes from up and downregulated genes with a p-value <0.01. F) Overlap between differentially regulated genes in the RNA-sequencing from E14.5 cap mesenchyme and kidney organoids. G) Number of differentially regulated genes belonging to the non-coding RNA class.

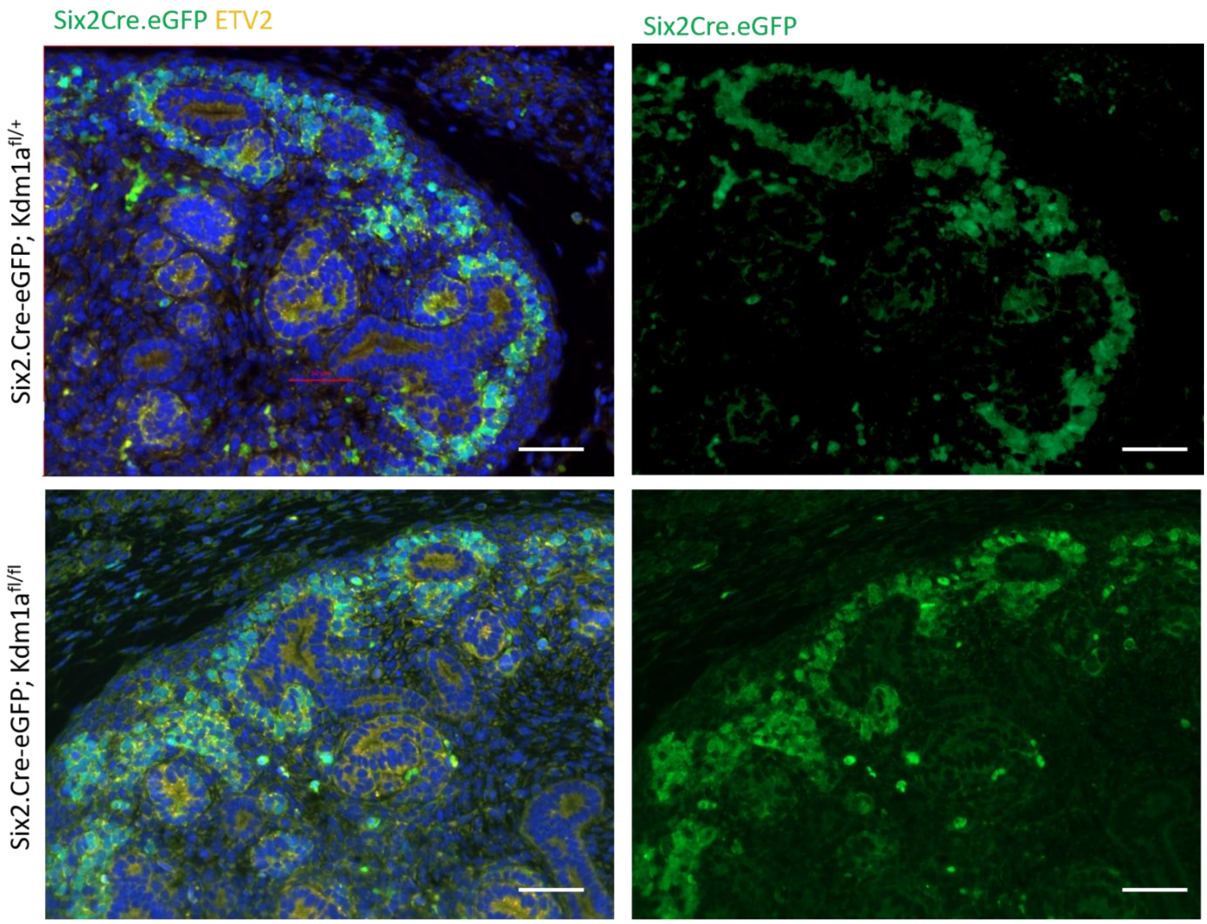

Supplementary Figure 6. Nephron progenitor cells at E14.5 kidneys. Immunofluorescence staining for ETV2 (orange) and endogenous Six2.Cre-eGFP (green) in embryonic day 14.5 (E14.5) kidneys from heterozygous (upper panel) and *Kdm1a* knockout (lower panel) mice. The amount of Six2.Cre-eGFP–positive nephron progenitor cells appear comparable between genotypes. Scale bars: 50 μm. Blue: DAPI staining of nuclei.

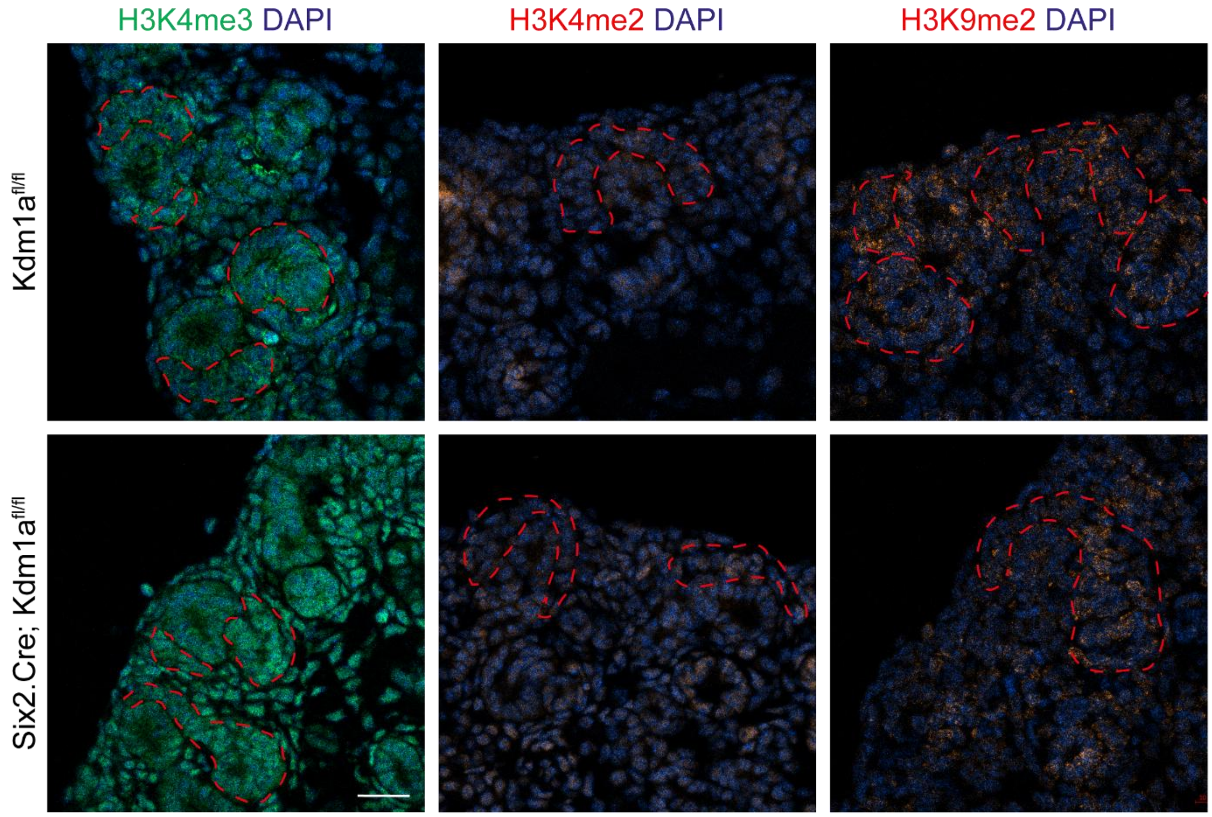

Supplementary Figure 7. Histone modifications in p0 kidneys. Immunofluorescence staining for histone marks H3K4me3 (green), H3K4me2 (red), and H3K9me2 (red) on kidney sections from postnatal day 0 (p0) wild-type (upper panels) and *Kdm1a* knockout (lower panels) mice. Red dotted lines outline the cap mesenchyme and budding nephrons. Scale bars: 20 µm.

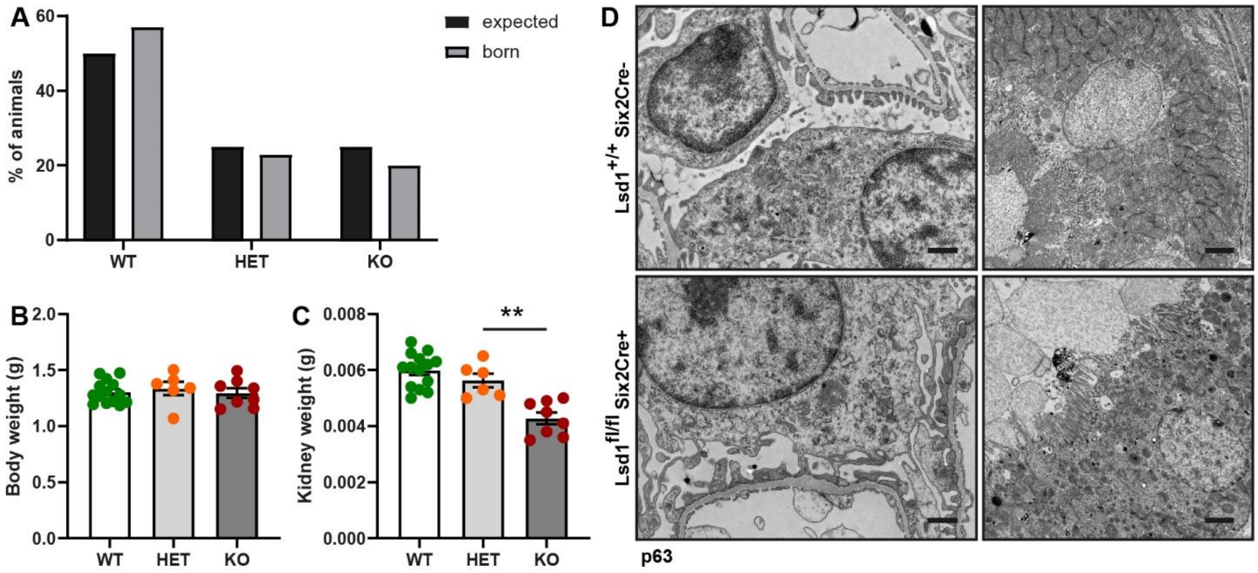

Supplementary Figure 8: Genotype and phenotype of the p0 and p63 *Kdm1a* KO animals. A) Genotypes of the pups from Six2Cre/+ *Kdm1a* fl/wt and Six2Cre/- *Kdm1a* fl/fl breedings followed the expected Mendelian ratio (expected - light grey, born - dark grey; wt, n = 80; het, n = 32; ko, n = 28). B) Body weight of newborn wildtype, heterozygous and *Lsd1* knockout pups was the same. C) Kidney weight of newborn *Kdm1a* knockout animals was reduced compared to wildtype and heterozygous litter mates. **, p=0.0012. D) Transmission electron microscopy of wildtype and *Kdm1a*/*Lsd1* KO kidneys at p63 shows foot process effacement and accumulation of cell debris in urinary space. Scale bars left panels, 750 nm, middle and right panels, 2 µm.

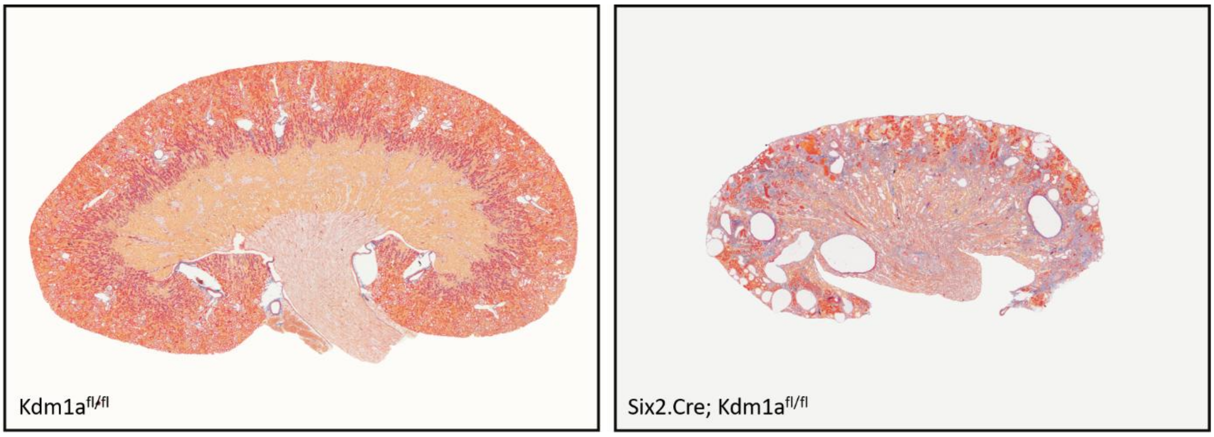

Supplementary Figure 9. SFOG staining of whole kidney sections at postnatal day 63. Whole kidney sections from wild-type (left panel) and *Kdm1a* knockout (right panel) mice were stained with SFOG (Sirius Red, Fast Green, Orange G) to assess tissue morphology and fibrosis.

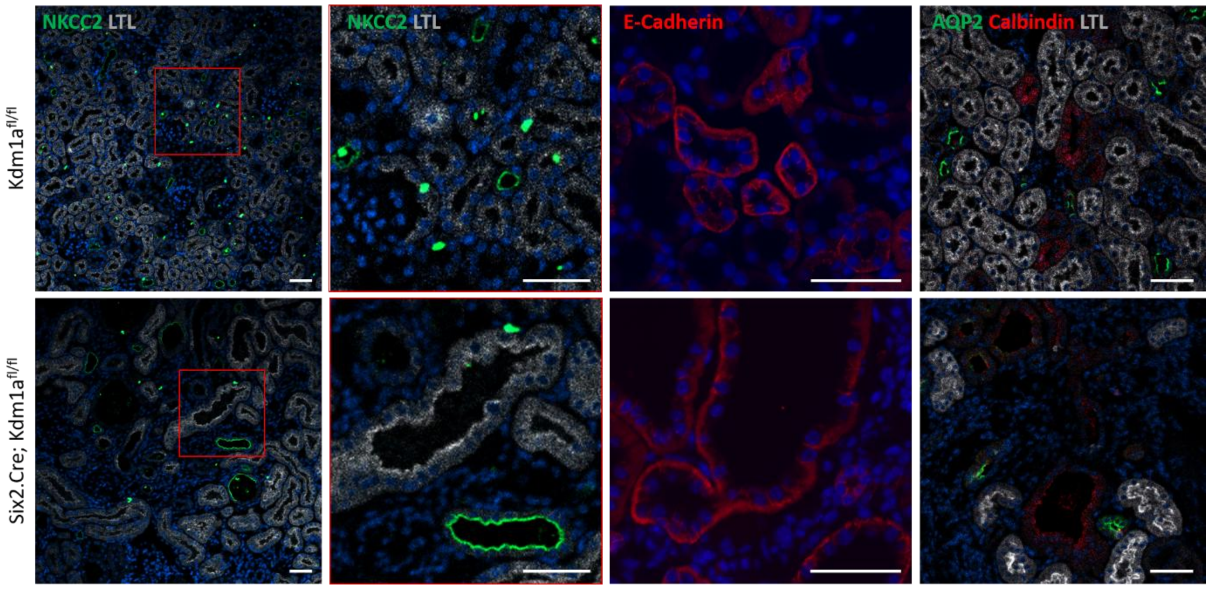

Supplementary Figure 10. Cyst formation in distinct tubular segments of p63 kidneys. Immunofluorescence staining for segment-specific tubular markers in postnatal day 63 (p63) kidney sections from wild-type (upper panels) and *Kdm1a* knockout (lower panels) mice. From left to right: proximal tubules (Lotus Tetragonolobus Lectin, LTL), thick ascending limb (NKCC2), distal tubules (E-cadherin), distal convoluted and connecting tubules (Calbindin), and collecting ducts (AQP2). Red squares indicate areas shown at higher magnification in adjacent panels. Scale bars: 20 µm.

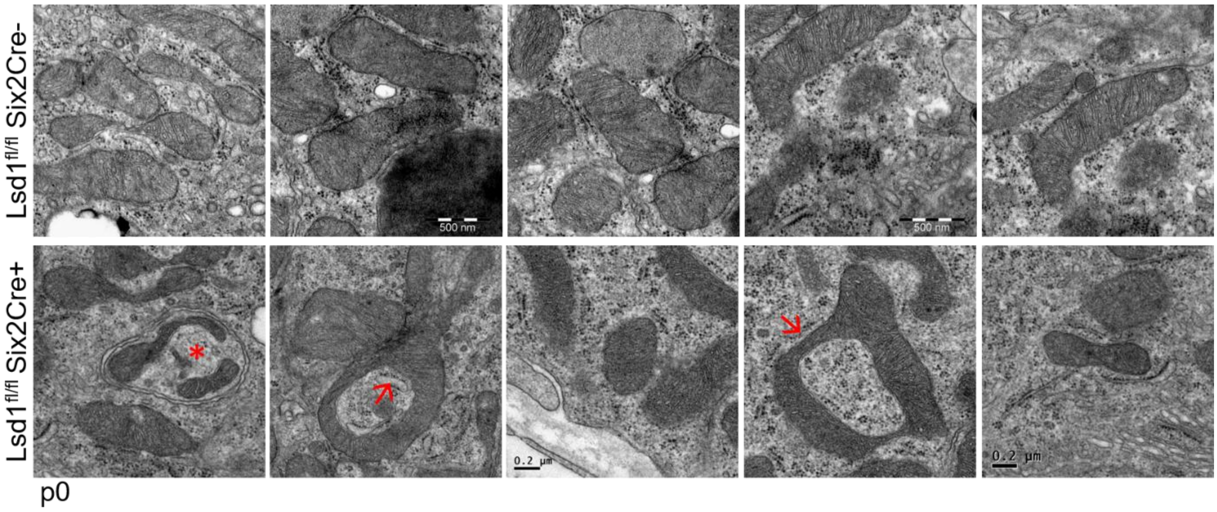

Supplementary Figure 11: Loss of *Kdm1a* results in irregular mitochondria. Transmission electron microscopy of wildtype (upper panel) and *Kdm1a* KO (lower panel) kidneys at p0 shows mitophagy (red asterisk) and mitochondrial fusion (red arrows) in the KO kidneys. Scale bars, upper panel, 500 nm, lower panel, 2 µm.

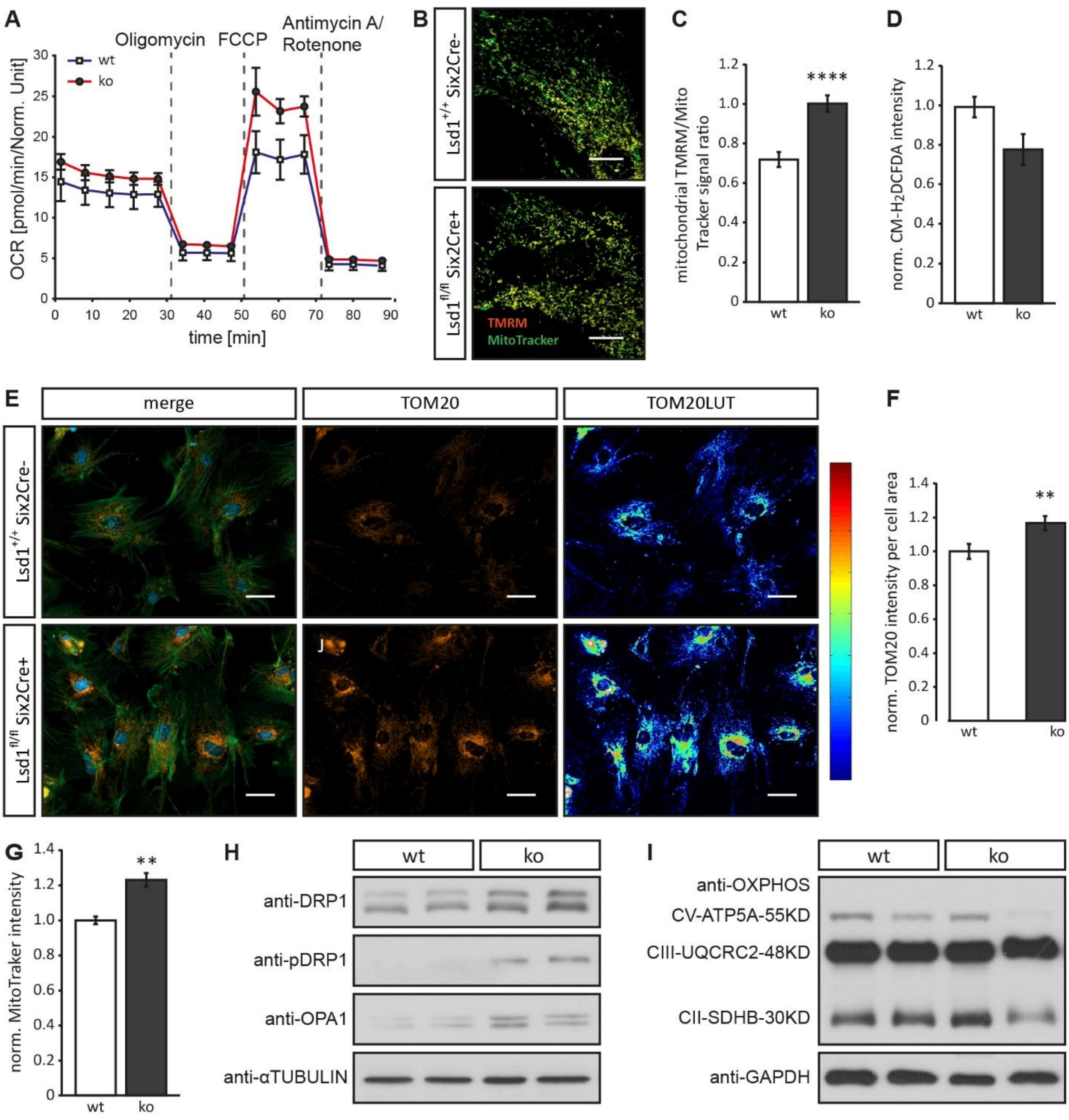

Supplementary Figure 12: Loss of *Kdm1a* leads to altered mitochondrial function. A) Oxygen consumption rate (OCR) determined by Seahorse Extracellular Flux Analyzer exhibited higher FCCP response of primary renal cells *Kdm1a* KO compared to wildtype cells. B) Immunofluorescence analysis of mitochondrial membrane potential using tetramethylrhodamine methyl ester (TMRM; red) and MitoTracker (green). Scale bars, 5 µm. C) Quantification of mitochondrial TMRM/MitoTracker signal ratio shows significant increase in mitochondrial membrane potential (normalized to mean KO signal ratio) (ctl, n = 21, N = 2; ko, n = 20, N = 2). D) The oxidative stress indicator CM-H2DCFDA intensity shows no increase of ROS by loss of LSD1 (normalized to mean WT intensity) (ctl, n = 6, N = 2; ko, n = 6, N = 2). E) Staining of mitochondrial membrane marker TOM20 (orange) (normalized to mean WT intensity per cell area) (ctl, n = 55, N = 2; ko, n = 51, N = 2) showed significant increase of normalized TOM20 intensity per cell area. Scale bars, 10 µm. F) Quantification of TOM20 intensity. G) Quantification of MitoTracker intensity also reveals very significant increase in mitochondrial signal

(normalized to mean WT intensity) (ctl, n = 6, n = 2; ko, n = 6, N = 2). H) Western blot analysis shows enrichment of mitochondrial-specific markers DRP1, pDRP1 and OPA1 in KO (n=2). I) Western blot analysis of OXPHOS-proteins CV-ATP5A, CIII-UQCRC2 and CII-SDHB reveals no alteration in KO (n=2).

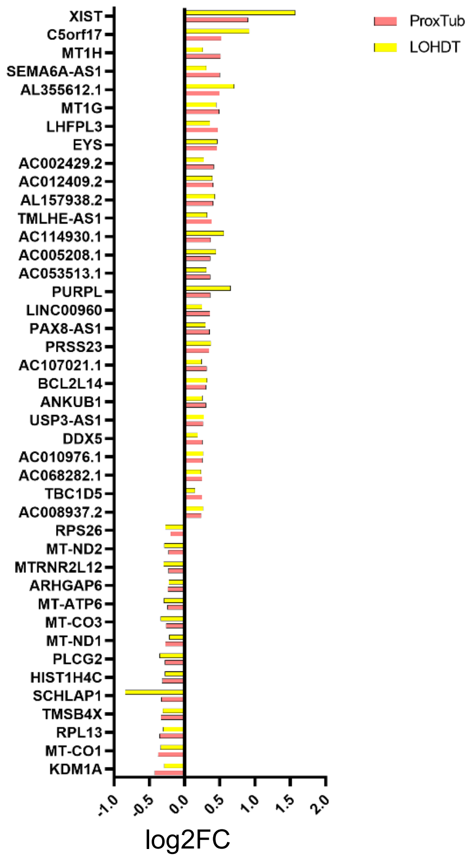

Supplementary Figure 13. Genes differentially expressed in both proximal tubules and loop of Henle/distal tubules. Log2 fold change shows upregulation of genes, many of them non-protein coding, such as XIST, PURPL, antisense (AS) genes and noncoding RNAs**.** Downregulated genes contain mitochondrial genes, PLCG2, TMSB4X and ARHGAP6.

A

WP Ciliary La	Offtarget	EC	MS1	MS2	NPClike	NPC	RV	POD	matPOD	PT	LOHDT	CD	UB
H3F3A	n.s.	n.s.	-0.1794186	n.s.	n.s.	n.s.	n.s.	-0.1877122	-0.283857	n.s.	n.s.	n.s.	n.s.
TFAP2B	0.58168985	n.s.	n.s.	n.s.	n.s.	n.s.	n.s.	n.s.	n.s.	n.s.	n.s.	n.s.	n.s.
CEP170	n.s.	n.s.	-0.1589923	n.s.	n.s.	n.s.	n.s.	n.s.	n.s.	n.s.	n.s.	n.s.	n.s.
CTBP2	n.s.	n.s.	n.s.	-0.2217731	n.s.	n.s.	n.s.	n.s.	n.s.	n.s.	n.s.	n.s.	n.s.
CREBBP	n.s.	n.s.	-0.1315911	n.s.	n.s.	n.s.	-0.2709795	-0.1573061	n.s.	n.s.	n.s.	n.s.	n.s.
CAMK2A	n.s.	n.s.	n.s.	n.s.	n.s.	n.s.	n.s.	-0.1402555	n.s.	n.s.	n.s.	n.s.	n.s.
ACSL3	n.s.	n.s.	n.s.	n.s.	n.s.	n.s.	n.s.	-0.141377	n.s.	n.s.	n.s.	n.s.	n.s.
EXOC6B	n.s.	n.s.	n.s.	n.s.	n.s.	n.s.	n.s.	-0.1403092	n.s.	n.s.	n.s.	n.s.	n.s.
EXOC6	n.s.	n.s.	n.s.	-0.2544213	n.s.	n.s.	n.s.	n.s.	n.s.	n.s.	n.s.	n.s.	n.s.
VIM	n.s.	n.s.	n.s.	n.s.	n.s.	n.s.	n.s.	-0.179748	n.s.	n.s.	n.s.	n.s.	n.s.
WDR26	n.s.	n.s.	-0.1326139	n.s.	n.s.	n.s.	n.s.	n.s.	n.s.	n.s.	n.s.	n.s.	n.s.
RMND5A	n.s.	n.s.	n.s.	-0.2146921	n.s.	n.s.	n.s.	n.s.	n.s.	n.s.	n.s.	n.s.	n.s.
DDX5	0.48991855	n.s.	n.s.	0.20717671	0.21983958	0.27375112	0.23945141	0.30356923	0.30686224	0.25652342	0.19182801	n.s.	0.26572105
MYL6	n.s.	n.s.	n.s.	n.s.	n.s.	n.s.	n.s.	-0.1287928	n.s.	n.s.	n.s.	n.s.	n.s.
IQGAP2	n.s.	n.s.	n.s.	n.s.	n.s.	n.s.	n.s.	-0.2288333	n.s.	n.s.	n.s.	n.s.	n.s.
SMC4	n.s.	n.s.	n.s.	n.s.	n.s.	n.s.	-0.2180715	n.s.	n.s.	n.s.	n.s.	n.s.	n.s.

B

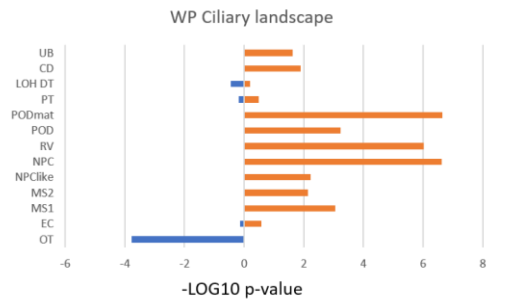

C

Ciliary genes	Offtarget	MS1	MS2	NPClike	NPC	RV	POD	mat POD	PT	LOH DT	CD	UB
PKD1/PKD2	n.s.	n.s.	n.s.	n.s.	n.s.	n.s.	n.s.	n.s.	n.s.	n.s.	n.s.	n.s.
PKHD1	n.s.	n.s.	n.s.	n.s.	n.s.	n.s.	n.s.	n.s.	0.342	n.s.	0.3999	n.s.
GLIS2	n.s.	n.s.	n.s.	n.s.	n.s.	n.s.	n.s.	n.s.	n.s.	n.s.	n.s.	n.s.
GLIS3	n.s.	0.226	n.s.	n.s.	n.s.	n.s.	n.s.	n.s.	n.s.	n.s.	n.s.	n.s.
CC2D2A	n.s.	n.s.	n.s.	n.s.	n.s.	n.s.	0.146	n.s.	n.s.	n.s.	n.s.	n.s.
VHL	n.s.	n.s.	n.s.	n.s.	n.s.	n.s.	n.s.	n.s.	n.s.	n.s.	n.s.	n.s.
NPHP1-9	n.s.	n.s.	n.s.	n.s.	n.s.	n.s.	n.s.	n.s.	n.s.	n.s.	n.s.	n.s.
NEK1	n.s.	n.s.	n.s.	n.s.	n.s.	n.s.	n.s.	n.s.	n.s.	n.s.	n.s.	n.s.
BBS3-14	n.s.	n.s.	n.s.	n.s.	n.s.	n.s.	n.s.	n.s.	n.s.	n.s.	n.s.	n.s.
MKS1/3	n.s.	n.s.	n.s.	n.s.	n.s.	n.s.	n.s.	n.s.	n.s.	n.s.	n.s.	n.s.
B9D1/2	n.s.	n.s.	n.s.	n.s.	n.s.	n.s.	n.s.	n.s.	n.s.	n.s.	n.s.	n.s.
TMEM67/107/216/231	n.s.	n.s.	n.s.	n.s.	n.s.	n.s.	n.s.	n.s.	n.s.	n.s.	n.s.	n.s.

Supplementary Figure 14. Ciliary gene dysregulation upon *KDM1A* knockout in renal organoids. A) Overlap of differentially expressed genes with the WikiPathways *Ciliary Landscape* gene set. B) Gene set enrichment analysis (GSEA) of the *Ciliary Landscape* pathway across individual cell populations. C) Additional cilia-associated genes and genes previously linked to cystic phenotypes.

Supplementary Tables:

Supplementary Table 2: Primers used for mouse genotyping analysis as well as generation of *in situ* probes.

Primer	Sequence
Cre Forward	5' GCA TTA CCG GTC GAT GCA ACG AGT GAT GAG 3'
Cre Reverse	5' GAG TGA ACG AAC CTG GTC GAA ATC AGT GCG 3'
Fabpi-200 Forward	5’ TGG ACA GGA CTG GAC CTC TGC TTT CCT AGA 3’
Fabpi-200 Reverse	5’ TAG AGC TTT GCC ACA TCA CAG GTC ATT CAG 3’
Kdm1a knockout Forward	5' CCT ACA CTG TGC CAG GCT GC 3'
Kdm1a knockout Reverse	5' GCA GGC GGT TTG AAA TGT ATT C 3'
Kdm1a knockin Forward	5’ CCA GCT GCT TGT TGG TGC 3’
Kdm1a knockin Reverse	5’ TGG AGT GAA GTG GTT ACC TGC 3’
Sry Forward	5’ TTG TCT AGA GAG CAT GGA GGG CCA TGT CAA 3’
Sry Reverse	5’ CCA CTC CTC TGT GAC ACT TTA GCC CTC CGA 3’
Tomato/EGFP Forward	5' CTC TGC TGC CTC CTG GCT TCT 3'

Tomato/EGFP Reverse (wt)	5' CGA GGC GGA TCA CAA GCA ATA 3'
Tomato/EGFP Reverse (mut)	5' TCA ATG GGC GGG GGT GCT T 3'
Kdm1a in-situ Forward	5' cgc ggg ACG CGT GCCCTGGTAGCAGGAGAAG 3'
Kdm1a in-situ Reverse	5' cgc ggg GCG GCC GCC AAGTTGCTGTCAGCCAAAGG 3'
Six2 in-situ Forward	5' cgc ggg ACG CGT CGG ACC CAC TGC AGC ATC ACC 3'
Six2 in-situ Reverse	5' cgc ggg GCG GCC GCC TTC AGG TGC TTC TGG GGT GCA G 3'

Supplementary Table 3: Antibodies used for immunofluorescence and western blot analysis.

**Primary antibodies**	**Dilution**	**Condition**	**Company**
goat anti-Megalin (P-20, sc-16478)	1:100	IF, pH9	Santa Cruz Biotechnology
rabbit anti-LSD1 (#20752)	1:100	IF, pH9	AG Schüle, Freiburg, Germany
mouse anti-GFP (B-2, sc-9996)	1:100	IF, pH9	Santa Cruz Biotechnology
rabbit anti-tom20 (sc-11415)	1:100	IF, pH9	Santa Cruz Biotechnology
guinea-pig anti-Nephrin (GP-N2)	1:200	IF, pH9	Progen
Lotus tetragonobulus lectin (B-1325)	1:100	IF, pH9	Vector Laboratories
rabbit anti-KDM1A (#3544)	1:1000	WB	AG Schüle, Freiburg, Germany
mouse anti-GAPDH (6C5, ab8245)	1:5000	WB	Abcam
rabbit anti-DRP1 (5391S)	1:1000	WB	Cell Signaling Technology
rabbit anti-pDRP1 (3455)	1:1000	WB	Cell Signaling Technology
mouse anti-OPA1 (612607)		WB	BD Biosciences
mouse anti-OXPHOS (ab110413)	1:3000	WB	Abcam
mouse anti-αTubulin (T 9026)	1:10000	WB	Sigma-Aldrich GmbH
mouse anti-H3K4me2 (39679, Clone MABI 0303)	1:100	IF, pH9	Active Motif
rabbit anti-H3K4me3 (G.532.8)	1:100	IF, ph9	Thermo Scientific
mouse anti-H3K9me2 (1220)	1:100	IF, ph9	Abcam

E-Cadherin Monoclonal Antibody (4A2C7)	1:100	IF, ph9	Life technologies
mouse anti-Calbindin (D-28k)	1:100	IF, ph9	StressMarq Biosciences
anti-AQP2 (AQP-002)	1:100	IF, ph9	Alomone Labs
anti-NKCC2 (SPC-401)	1:100	IF, ph9	StressMarq Biosciences
rabbit anti-ETV2 (ab181847)	1:100	IF, pH9	Abcam
**Secondary antibodies/fluorescent compounds**	**Dilution**	**Condition**	**Company**
Alexa Fluor 488 donkey anti-goat IgG	1:500	IF, pH9	Thermo Fisher Scientific
Alexa Fluor 488 donkey anti-mouse IgG	1:500	IF, pH9	Thermo Fisher Scientific
Alexa Fluor 555 donkey anti-rabbit IgG	1:500	IF, pH9	Thermo Fisher Scientific
Alexa Fluor 488 Phalloidin	1:1000	IF, pH9	Thermo Fisher Scientific
Hoechst	1:5000	IF, pH9	Molecular Probes
DAPI	1:5000	IF, pH9	Sigma Aldrich
goat anti-rabbit IgG-HRP (#7074S)	1:3000	WB	Cell Signaling Technology
goat anti-mouse IgG-HRP (#P0447)	1:10000	WB	Dako

Supplementary Table 4: Primers used for the generation of CRISPR/Cas9 KO iPSCs.

Oligo	Forward	Reverse
Guide RNA	GGCAAGGCTTTTCGGACCCACGG	GGCGGTGTCGTTTGAGGGAAGGG
Genotyping Exon1_external	AGCTACACGTTCTTTGCTGC	GTCAACACCGGCAAAGACTT
Genotyping Exon1_internal	TTATCTGGGAAGAAGGCGGC	CCGGAGTCTCTGCTATTCCA
Genotyping Exon1_2	TGGAATAGCAGAGACTCCGG	CACGTCAGGGAGCCATTTTC

Genotyping Exon1_3	AGCTACACGTTCTTTGCTGC	GCCGCCTTCTTCCCAGATAA
Sequencing primer	GCATGCAAACCCGAAAGTCC	
Offtarget 1	CCATAGCTCCAGACTACGCA	GGTATTCCCCTCCAACCCAG
Offtarget 2	GGGACTATGGCTAAGGGGTC	TGTACCGAAAGCACAGGGAT
Offtarget 3	CACCCTGTCCATGAAGAGGT	GCACTTCAGCTTCCTCAAGG
Offtarget 4	AGGAGAAAGTCGTTGCAGGA	CTCCGAGATTTGTCCCTGGT
Offtarget 5	AACAGGAATGAGAGGCCACA	CAGGCAAGAAAGGAGCGTTT
Offtarget 6	AGTCCTTTCCAGCCTTACCG	CACCCTTTTCTTCGCTTGCT
Offtarget 7	CCTGACACTGAGCACGTTTC	TCCCAGCTGCAATCTCTGAA
Offtarget 8	TGATGTGGGAGCATGGAAGT	TGAGGGTGTTCATGCCTGAT
Offtarget 9	TGGGGATGGAGATGGAGTTG	GAACAAGGCAGGAAAGTGGG
Offtarget 10	AGAAGGAACCTGGAGAGCAG	AGGAAGAGAGCCAAGGACAC

Supplementary References

1. Bolger AM, Lohse M, Usadel B. Trimmomatic: a flexible trimmer for Illumina sequence data. Bioinformatics. 2014 Aug 1;30(15):2114-20. doi: 10.1093/bioinformatics/btu170. Epub 2014 Apr 1. PMID: 24695404; PMCID: PMC4103590.

2. Dobin A, Davis CA, Schlesinger F, Drenkow J, Zaleski C, Jha S, Batut P, Chaisson M, Gingeras TR. STAR: ultrafast universal RNA-seq aligner. Bioinformatics. 2013 Jan 1;29(1):15-21. doi: 10.1093/bioinformatics/bts635. Epub 2012 Oct 25. PMID: 23104886; PMCID: PMC3530905.

3. Love MI, Huber W, Anders S. Moderated estimation of fold change and dispersion for RNA-seq data with DESeq2. Genome Biol. 2014;15(12):550. doi: 10.1186/s13059-014-0550-8. PMID: 25516281; PMCID: PMC4302049.

4. Zeisel A, Hochgerner H, Lönnerberg P, Johnsson A, Memic F, van der Zwan J, Häring M, Braun E, Borm LE, La Manno G, Codeluppi S, Furlan A, Lee K, Skene N, Harris KD, Hjerling-Leffler J, Arenas E, Ernfors P, Marklund U, Linnarsson S. Molecular Architecture of the Mouse Nervous System. Cell. 2018 Aug 9;174(4):999-1014.e22. doi: 10.1016/j.cell.2018.06.021. PMID: 30096314; PMCID: PMC6086934.
