# Supplementary material for: Lysine-specific histone demethylase 1a regulates nephron development and long-term transcriptional programming
Source: JCI Insight. 2026 Mar 9;11(5):e190283. doi: 10.1172/jci.insight.190283 (PMC13041676; doi:10.1172/jci.insight.190283)
Supplement: Unedited blot and gel images [file jciinsight-11-190283-s234.pdf]

Figure 1F

Anti-KDM1A

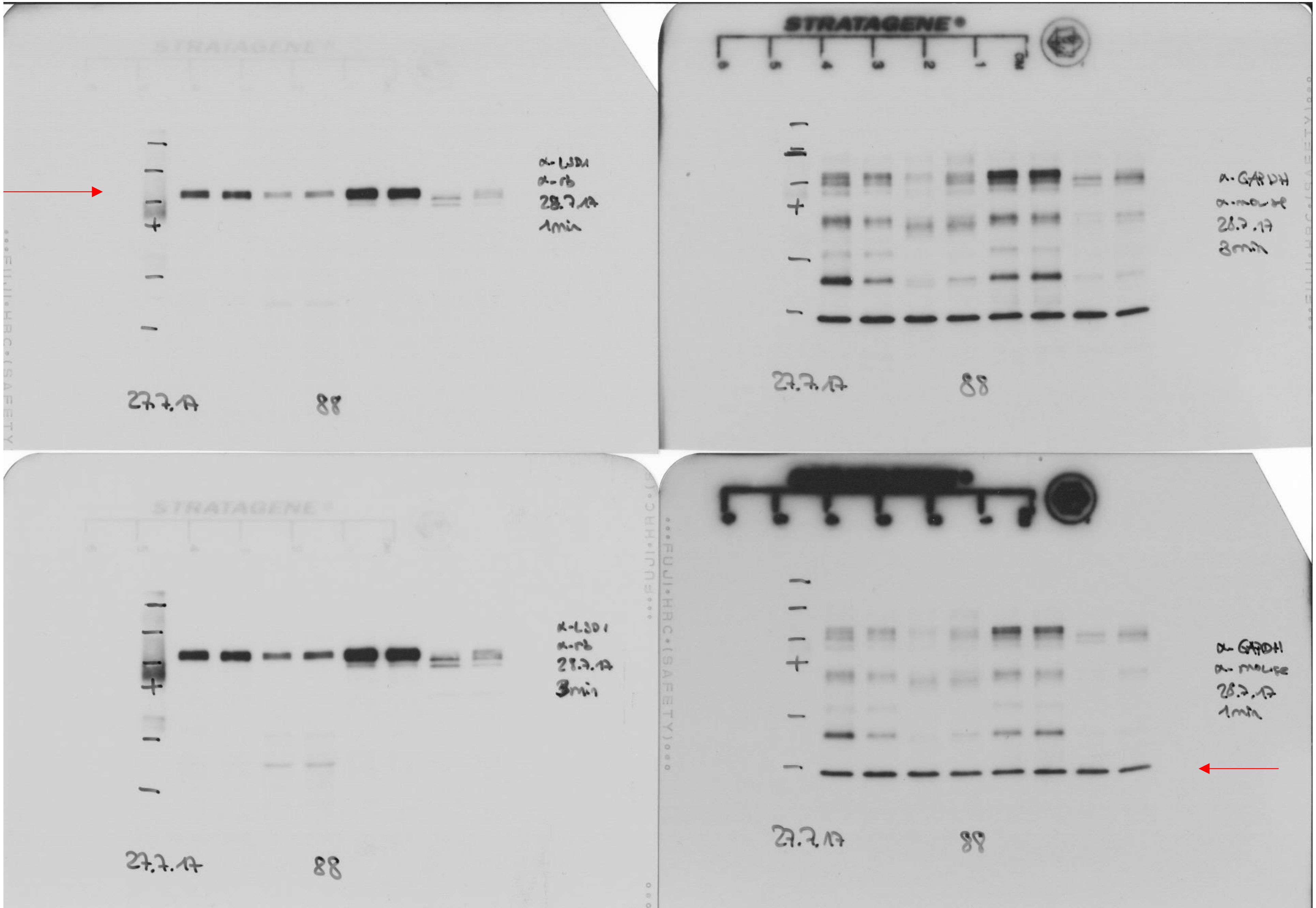

Anti-GAPDH

Figure 6C

Celllysate: buffer RIPA

Anti-KDM1A

Anti-beta actin

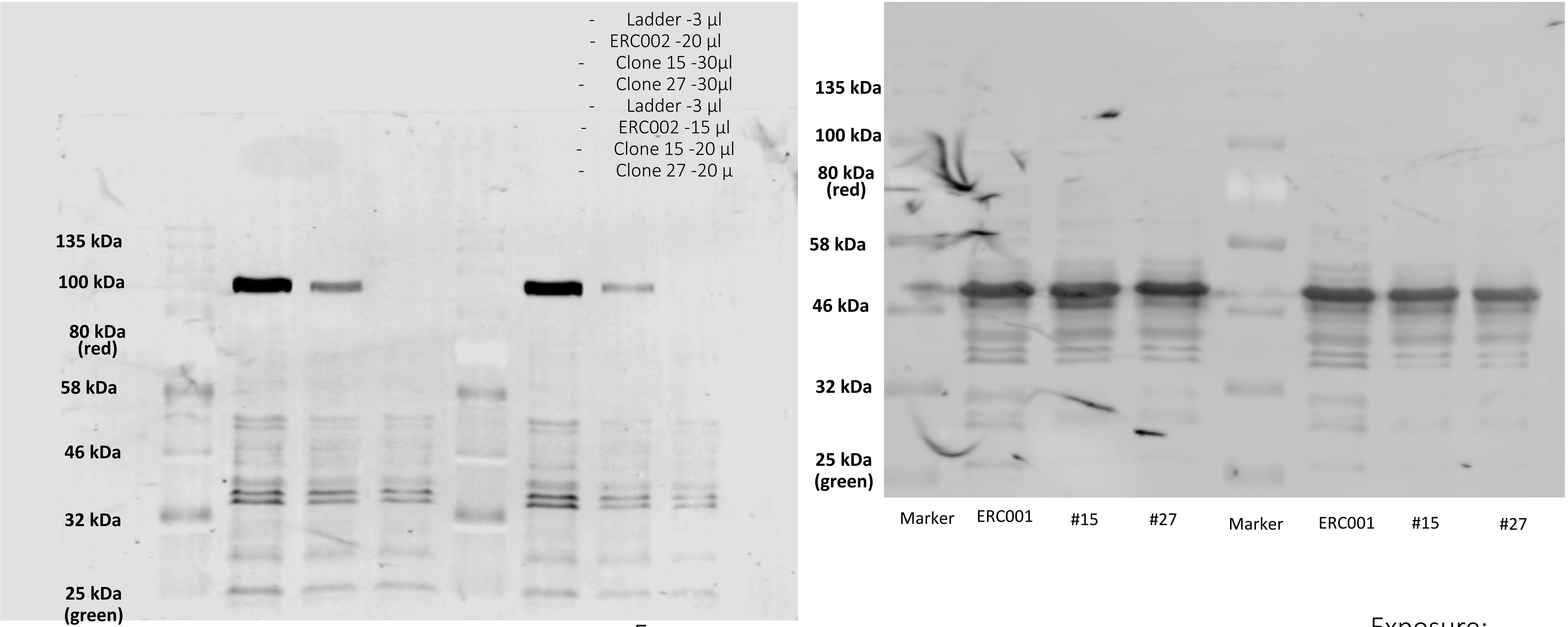

Exposure:

- 800nm, lowest quality, f=0.0mm, 169µm

Exposure:

- 700nm, lowest quality, f=0.0mm, 169µm
